# Disentangling enforced and voluntary behaviour change during the COVID-19 Pandemic, the Netherlands, 2020 to 2023

**DOI:** 10.2807/1560-7917.ES.2026.31.44.2600078

**Published:** 2026-11-05

**Authors:** Carlijn Bussemakers, Federica Giardina, Marijn de Bruin

**Affiliations:** 1 IQ Health science department, Radboud university medical centre, Nijmegen, The Netherlands; 2 Department Behaviour and Health, Centre for Prevention, Lifestyle and Health, National Institute for Public Health and the Environment (RIVM), Bilthoven, The Netherlands

**Keywords:** Infectious disease outbreak, behaviour change, restrictive measures, adherence, compliance, public health policy

## Abstract

**BACKGROUND:**

Effective pandemic mitigation relies on population behaviour change. To understand how to achieve this, it is important to not only consider restrictive policies that enforce behaviour change but also consider voluntary responses to changes in pandemic severity and behavioural advice.

**AIM:**

The current study investigates to what extent change in protective behaviour during the COVID-19 pandemic in the Netherlands can be attributed to self-guided behaviour change (in response to changes in pandemic severity), adherence to government behavioural advice, or compliance with restrictive policies.

**METHODS:**

We analysed survey and mobility data on seven protective behaviours from 2020 to 2023. Using time-series and mixed-effects models, we examined associations between pandemic severity (hospitalisations), government behavioural advice, and restrictive policies with these behaviours. Control variables included seasonality, pandemic duration and demographic characteristics.

**RESULTS:**

Pandemic severity was consistently associated with increases in protective behaviour, while the impact of behavioural advice and restrictive policies varied. Moreover, the estimated influence of behavioural advice and restrictive policies was substantially reduced when including pandemic severity in the models.

**CONCLUSION:**

The consistent association between pandemic severity and protective behaviours indicates that people changed their behaviour in order to protect themselves and others, even without government advice or restrictive measures. Behaviour change could be further promoted through behavioural advice and/or restrictive policies, although the estimated impact of these approaches varied substantially between target behaviours. These results highlight the importance of voluntary behaviour change and accounting for type of behaviour change when designing and evaluating mitigation policies.

**Key public health message**

**What did you want to address in this study?**

During infectious disease outbreaks, mitigation efforts typically aim to reduce transmission through behaviour change. We wanted to understand how much protective behaviour during the COVID-19 pandemic in the Netherlands was self-guided (in responses to changes in pandemic severity) vs driven by government behavioural advice or enforced restrictive measures.

**What have we learnt from this study?**

We found consistent evidence of self-guided protective behaviour: people consistently changed their behaviour in response to worsening pandemic severity, also in the absence of enforced restrictive measures. The effects of government behavioural advice and restrictive policies varied by behaviour type and effects were typically overestimated if voluntary responses were not accounted for.

**What are the implications of your findings for public health?**

It is important to support and facilitate voluntary behaviour change during infectious disease outbreaks. In some instances, behaviour change could be further promoted through restrictive policies. Research into the effectiveness of pandemic policies should include measures of voluntary behaviour change.

## Introduction

The COVID-19 pandemic has highlighted the critical role of protective behaviour in controlling infectious disease transmission. Public health and social measures (PHSMs), which aim to promote this behaviour, can only reduce transmission to the extent that the public adheres to them. Consequently, public health surveillance must extend beyond tracking infection trends to also monitor how people respond behaviourally, both to changes in pandemic severity and to government measures.

Previous work has not been able to distinguish the influences of these contextual factors. Many studies have linked mobility changes to restrictive government policies [1-3], but since these policy restrictions co-occur with higher pandemic severity, it remains unclear whether restrictive policies caused this behaviour change. Because behaviour change does not only occur through government restrictions, it is important to also consider non-enforced (which we will refer to as ‘voluntary’) behaviour change [1,4,5]. Behavioural science research focusing on individual-level behaviour change indicates that voluntary behaviour change during a pandemic can manifest in two ways [4,6]. People adhered to non-enforced behavioural advice on for instance hand hygiene and keeping distance from others when they regarded these behaviours as feasible and when they believed these behaviours helped to protect themselves and others. But even in the absence of (changes in) the government’s behavioural advice, people increased their protective behaviour when the pandemic situation became more severe in order to protect themselves and others (self-guided protective behaviour). Although these studies shed light on the possible individual-level mechanisms of behaviour change, they were not able to distinguish the contextual influences of changes in restrictive policies (compliance), behavioural advice (adherence) and pandemic severity (self-guided behaviour change). Distinguishing these contextual influences is vital for understanding the true impact of government responses and for designing effective and acceptable mitigation strategies. Failure to account for voluntary behaviour change risks overestimating the effects of restrictive measures and underestimating the public's capacity for voluntary protective behaviour [7-10].

The current, preregistered study employs longitudinal behavioural data collected throughout the entire COVID-19 pandemic in the Netherlands to investigate to what extent behaviour change was associated with changes in pandemic severity (self-guided behaviour change), changes in adherence to behavioural advice or changes in compliance to restrictive policies. By focussing on national-level trends in specific forms of mobility as well as other forms of protective behaviour such as hygiene and testing behaviour, we could estimate associations with pandemic severity as well as with behaviour-specific advice and restrictive measures. Furthermore, the distinction enabled us to investigate the possible signalling function that restrictive policies had on protective behaviour that was not directly targeted by these policies.

Using such longitudinal data also has the advantage of allowing us to investigate to what extent the behaviour change associated with pandemic severity reduced over time as a result of risk habituation. Note that this phenomenon differs from ‘pandemic fatigue’, which refers to a possible overall reduction in protective behaviour over time. Previous research has criticised the notion of fatigue as lacking a theoretical foundation [11], and studies that controlled for factors such as changes in pandemic severity have found no evidence for a fatigue effect [4,8,12]. Risk habituation could be a more useful concept, as it refers to the phenomenon where people respond less strongly to a threat, such as the severity of a pandemic, the longer they are exposed to it [8,11].

We test the four hypotheses as listed in the Box.

Box **Hypotheses about the drivers of behavioural change during the COVID-19 pandemic, the Netherlands, 2020–2023**

1. People changed their protective behaviour in response to changes in pandemic severity (self-guided protective behaviour), government’s behavioural advice (adherence) and restrictive policies (compliance).

2. The estimated impact of behavioural advice and restrictive policies on preventive behaviour becomes smaller when accounting for pandemic severity (self-guided protective behaviour).

3. Restrictive policies have a signalling function and therefore also lead to an increase in protective behaviours that are not enforced (general enforcement).

4. The impact of pandemic severity on behaviour change becomes smaller over time.

## Methods

The preregistration of the hypotheses and analytical strategy of this study as well as the analytical dataset can be found on the Open Science Framework (https://osf.io/7d3mn). The section below discusses the main aspect of the data and methods, while Supplement part 1 provides a detailed description of the data sources and operationalisations. We deviated from the preregistration in one aspect: initially, we hypothesised a reduction in the impact of behavioural advice and restrictive policies over time, but because these were implemented during limited time periods, it was not possible to estimate this interaction.

### Study design

This study used observational data on protective behaviour during the COVID-19 pandemic in the Netherlands to assess to what extent changes in behaviour can be explained by changes in restrictive policies, behavioural advice and pandemic severity. Distinguishing these influences requires reliable, high frequency behavioural data covering different phases of the pandemic and a broad set of protective behaviours. We retrieved behavioural data from two sources: aggregated behavioural survey data collected by the Dutch National Institute for Public Health and the Environment (RIVM) between April 2020 and December 2023 and registered daily mobility data calculated by Google between February 2020 and October 2022 (962 days) [13]. The survey data were collected through both a repeated cross-sectional, nationally representative trend study (39 rounds, 199,363 observations) and a dynamic cohort study (21 rounds, 1,077,638 observations); detailed descriptions of these studies and the measurement properties of the behavioural measures have been published elsewhere [14,15].

### Variables

As outcome variables, we used seven measures of population-level protective behaviour. Four behaviours were measured using the survey data: daily handwashing frequency, avoiding at-home visits with friends and family, keeping 1.5 m distance during these at-home visits with friends and family, and testing when symptomatic. Three behaviours were measured using the Google mobility data: working from home, recreational mobility (visits to non-essential shops, the hospitality sector and the cultural sector) and visits to essential shops (supermarkets and pharmacies). These behaviours were selected because they were central in the Dutch COVID-19 response and they were specifically targeted by behavioural advice, restrictive policies or both.

Four predictor variables were used to test our hypotheses. Pandemic severity was measured using COVID-19 hospitalisations in the week before behaviour was observed [16]. Hospitalisation numbers were available throughout the entire pandemic and widely communicated to the public. We chose a 1-week lag for our main analyses to account for the time between rising infections, public communication and behaviour change. We also conducted several sensitivity checks with varying time lags between hospitalisations and behaviour to account for a possible delay between changes in pandemic severity and perceived severity. Government response was a categorical variable indicating the behaviour-specific restrictive policies and advice from the Dutch government that were in place at the time behaviour was measured. It was measured using a categorical variable with each level describing a (mutually exclusive) combination of policies and advice in place, ordered from least severe (advice) to most severe (enforced measures). In addition, we measured general enforcement as the sum of all restrictive policies that were in place, based on information from the COVID-19 Government response tracker [17]. Pandemic duration was measured as the number of days that had passed since the first confirmed COVID-19 case in the Netherlands (27 February 2020).

### Stepwise models

We used a stepwise modelling approach to test our hypotheses; the order is presented in the Table. We started with baseline models that included either only pandemic severity or government response, but our main interest lay in Model 2 which included the controlled effects of these factors to test Hypotheses 1 and 2. By including these factors in the same model, we could disentangle the variance in behaviour that can be attributed to either factor. General enforcement was added in Model 3 to test Hypothesis 3, while Model 4 included the interaction between pandemic severity and time to test Hypothesis 4. All hypotheses were tested with an alpha level of 0.05, and results are displayed using 95% confidence intervals (Cis). Because the Google mobility and behavioural survey data required different statistical models and confounders, we discuss these separately below.

**Table:** Stepwise modelling to test hypotheses about the drivers of behavioural change during the COVID-19 pandemic, the Netherlands, 2020–2023

Model	Predictor variables	Outcome variables	Hypotheses
1a: Baseline	• Pandemic severity	All	Baseline for H2
1b: Baseline	• Government response (behavioural advice and restrictive policies)	Survey data (handwashing, testing, visiting and distancing). Not possible for mobility data due to the feedback loop from behaviour to severity (see below).	Baseline for H2
2: Controlled effects	• Pandemic severity • Government response (behavioural advice and restrictive policies)	All	H1: People increase their protective behaviour in response to (i) pandemic severity (self-guided protective behaviour), (ii) government’s behavioural advice (adherence) and (iii) restrictive policies (compliance). H2: The estimated impact of behavioural advice and restrictive policies on preventive behaviour (adherence and compliance) becomes smaller when accounting for pandemic severity (self-guided protective behaviour).
3: With general enforcement	• Pandemic severity • Government response (behavioural advice and restrictive policies) • General enforcement	Handwashing and testing behaviour. For the other behaviours, behaviour-specific government response and general enforcement were too strongly correlated, introducing multicollinearity to the models.	H3: Restrictive policies have a signalling function and general enforcement therefore also leads to an increase in protective behaviours that are not enforced.
4: With interaction with duration	• Pandemic severity • Government response (behavioural advice and restrictive policies) • General enforcement • Interaction of duration with pandemic severity	All	H4: The impact of pandemic severity on behaviour change becomes smaller over time

H1–4: Hypothesis 1–4.

### Statistical models: survey data

Since the focus of this study was on population-level behaviour change, we aggregated the survey data on individual-level behaviour to measure population-level behaviour at each survey date. Aggregation was done by estimating generalised mixed models that included random intercepts at participant- and date- (survey round) level and fixed effects of date and sociodemographic factors (gender, age, education and employment) to account for possible differences in the sample composition between survey rounds. From these models, we derived predicted values of each behaviour for every survey date, representing population-level behavioural change over time.

We then analysed these predicted values in regression models to assess the impact of the main predictors. Linear regression for the continuous variable handwashing and logistic regression for the dichotomous variables avoiding visits, distancing and testing behaviour. A categorical predictor distinguishing the trend study from the cohort study was included as a control, to account for possible differences in behaviour between the samples of these studies. All continuous predictor variables were standardised. Results are reported as regression coefficients for continuous outcomes (handwashing) and as average marginal effects for the dichotomous outcomes and the interaction effect between hospitalisations and duration.

### Statistical models: mobility data

Google mobility outcomes were observed daily at the population level, making them more sensitive to seasonal changes and possible feedback loops. We therefore modelled the impact of pandemic severity and government response on mobility behaviour with bivariate time-series models using RStan. Each model included two regression equations: one for mobility and one for hospitalisations (capturing feedback from behaviour to severity). To control for seasonality, these models included day of week, school holidays, public holidays and pre-holiday effects. Models for work mobility also controlled for school closures. A detailed description of the model equations, priors, diagnostics plots and posterior probability checks can be found in Supplement part 2 with posterior analyses. Similar to the survey data analyses, all continuous variables were standardised. Model 1b was not considered for the mobility data, because excluding hospitalisations would mean the feedback loop with the effect of behaviour on pandemic severity would also be excluded. Without this feedback loop, it cannot serve as a baseline for Model 2 (Table).

### Sensitivity and exploratory analyses

We also performed five preregistered sensitivity analyses and one preregistered exploratory analysis. Sensitivity analyses included (i) removing the holiday period, (ii) changing the time lag between hospitalisations and behaviour to account for a possible early behavioural impact of rising infections, (iii) estimating a separate impact of pandemic severity due to the SARS-CoV-2 Omicron wave and (iv) employing different measures of testing behaviour. For the mobility models, we only considered sensitivity analysis (ii), as holidays were included as control variables in the main mobility models and the mobility data did not include the Omicron wave. We also explored the possible role of advice or requirements to wear facemasks in indoor public settings on recreational mobility and visits to essential shops.

## Results

Trends in behaviour change during the COVID-19 pandemic alongside changes in pandemic severity and government response are presented in Figures 1 and 2. Figure 1 shows that while there was very little change in handwashing behaviour, the other protective behaviours increased in periods with increased hospitalisations and/or the presence of the government’s behavioural advice or restrictive policies. Figure 2 shows that there are also clear drops in mobility under these circumstances, alongside other seasonal changes in mobility (day of week and holiday effects). Because increases in hospitalisations often coincided with the implementation of behavioural advice and/or restrictive policies, the regression and time-series models are needed to investigate their association with behaviour change.

**Figure 1:**
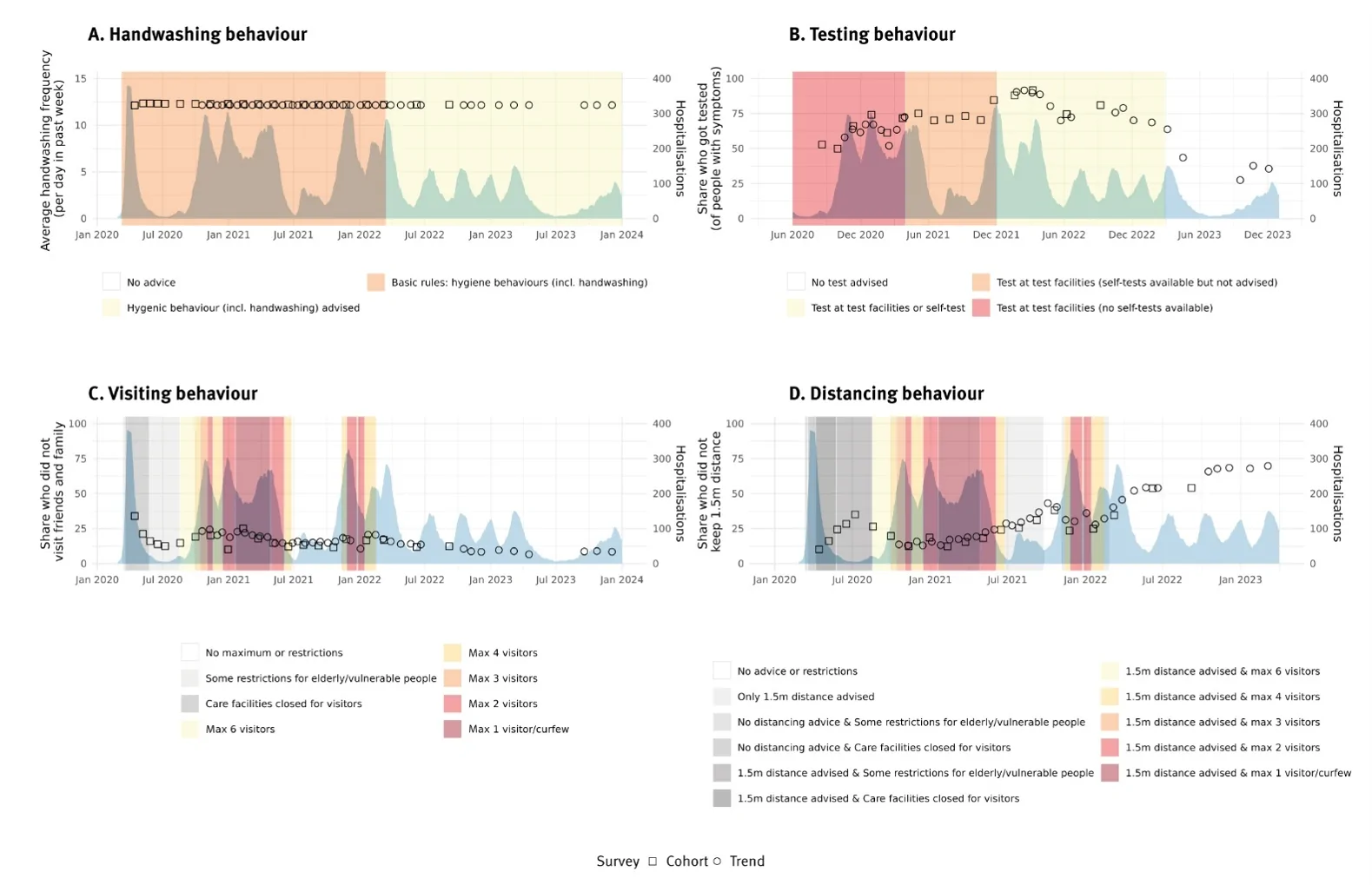
Descriptive trends in protective behaviour among the population (survey data), pandemic severity and government response^a^, the Netherlands, 2020–2023 (n = 60 survey data points)

^a^ Hygienic behaviours including handwashing were advised during the entire pandemic: initially they were framed as ‘basic rules’ (not enforced) and later they were eased to ‘advice’.

**Figure 2:**
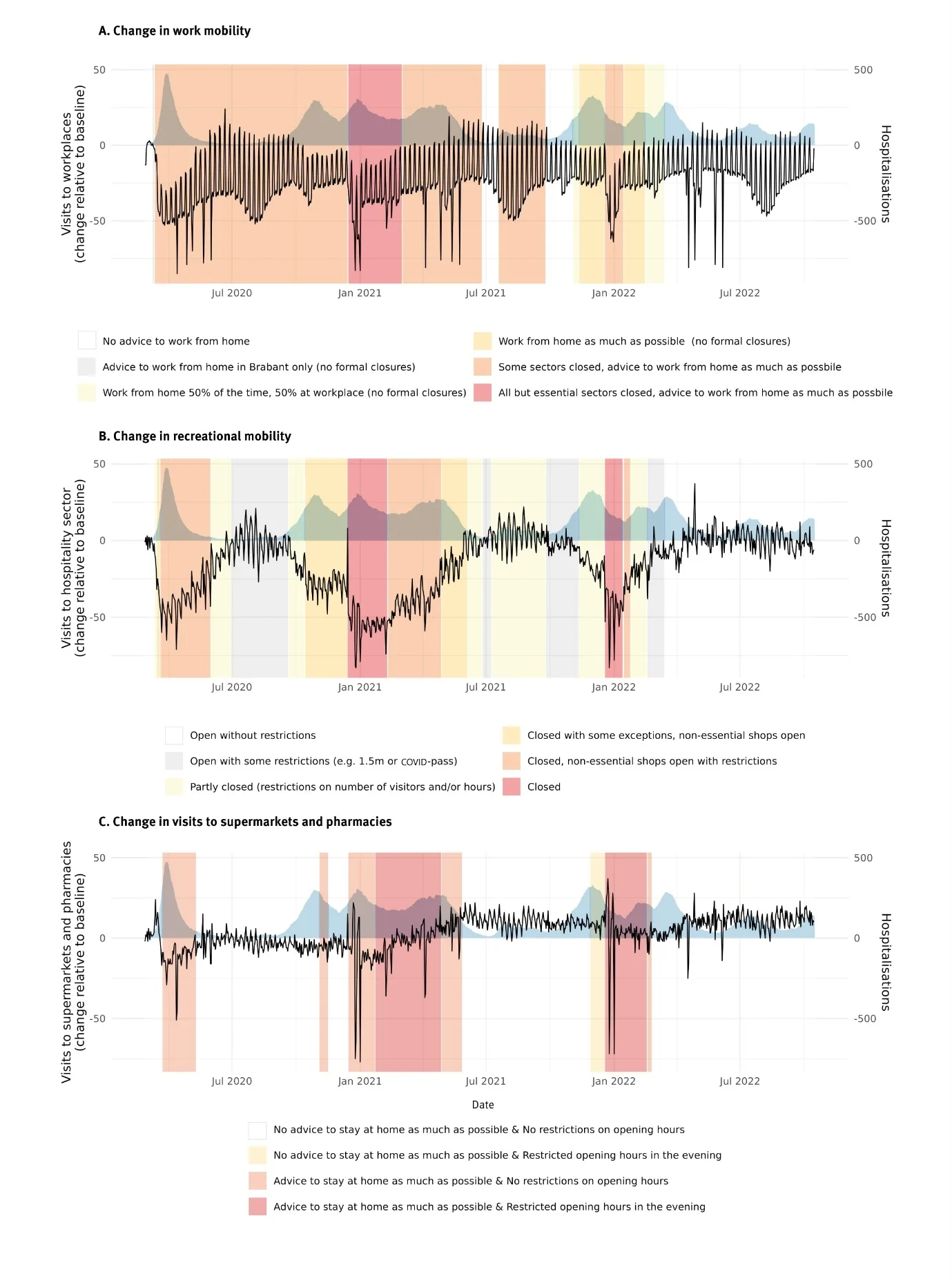
Descriptive trends in mobility behaviour among the population (mobility data) by pandemic severity and government response, the Netherlands, 2020–2022 (n = 962 mobility data points)

Figures 3 and 4 show the model coefficients of hospitalisations, behavioural advice and restrictive policies on behaviour. In line with the descriptive results, the regression coefficients in Figure 3 for handwashing (all models) confirm that there were only very small and mostly non-significant associations between hospitalisations, behavioural advice and general enforcement on handwashing behaviour. For the other behaviours, Figures 3 and 4 indicate larger associations with these factors. Both the baseline Model 1a and the controlled-effects Model 2 show that higher levels of hospitalisations were associated with higher rates of testing and lower rates of visiting, higher rates of distancing from friends and family (average marginal effects up to 0.08 for 1 standard deviation (SD) increase in hospitalisations), and all three forms of mobility (linear effects up to −0.25 for 1 SD increase in hospitalisations). However, the associations with visiting behaviour and working from home were small (Model 2).

**Figure 3:**
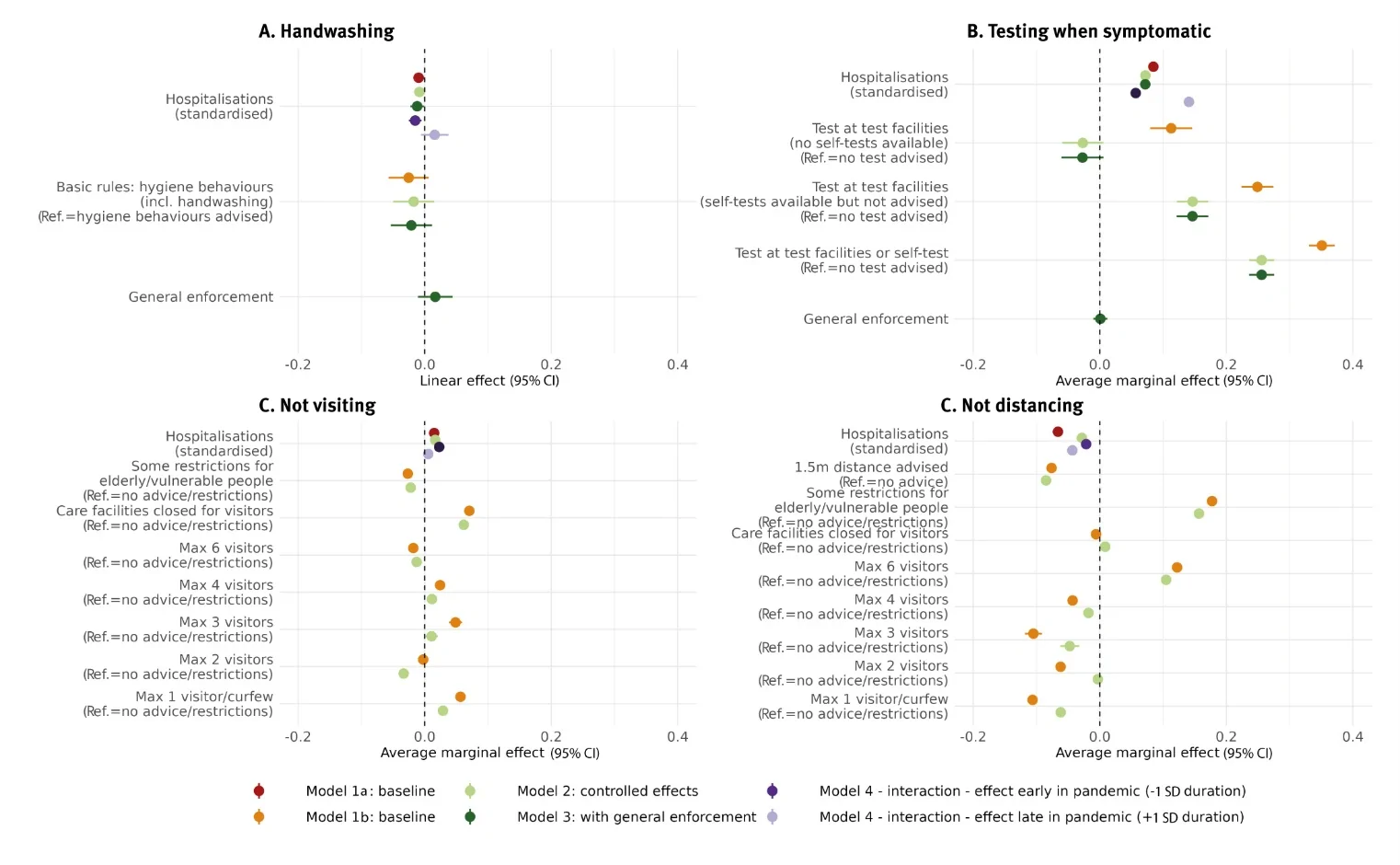
Regression coefficients and average marginal effects of pandemic severity and government response on behaviour in the population (survey data), the Netherlands, 2020–2023 (n = 60 survey data points)

CI: confidence interval; Ref: reference category; SD: standard deviation.

**Figure 4:**
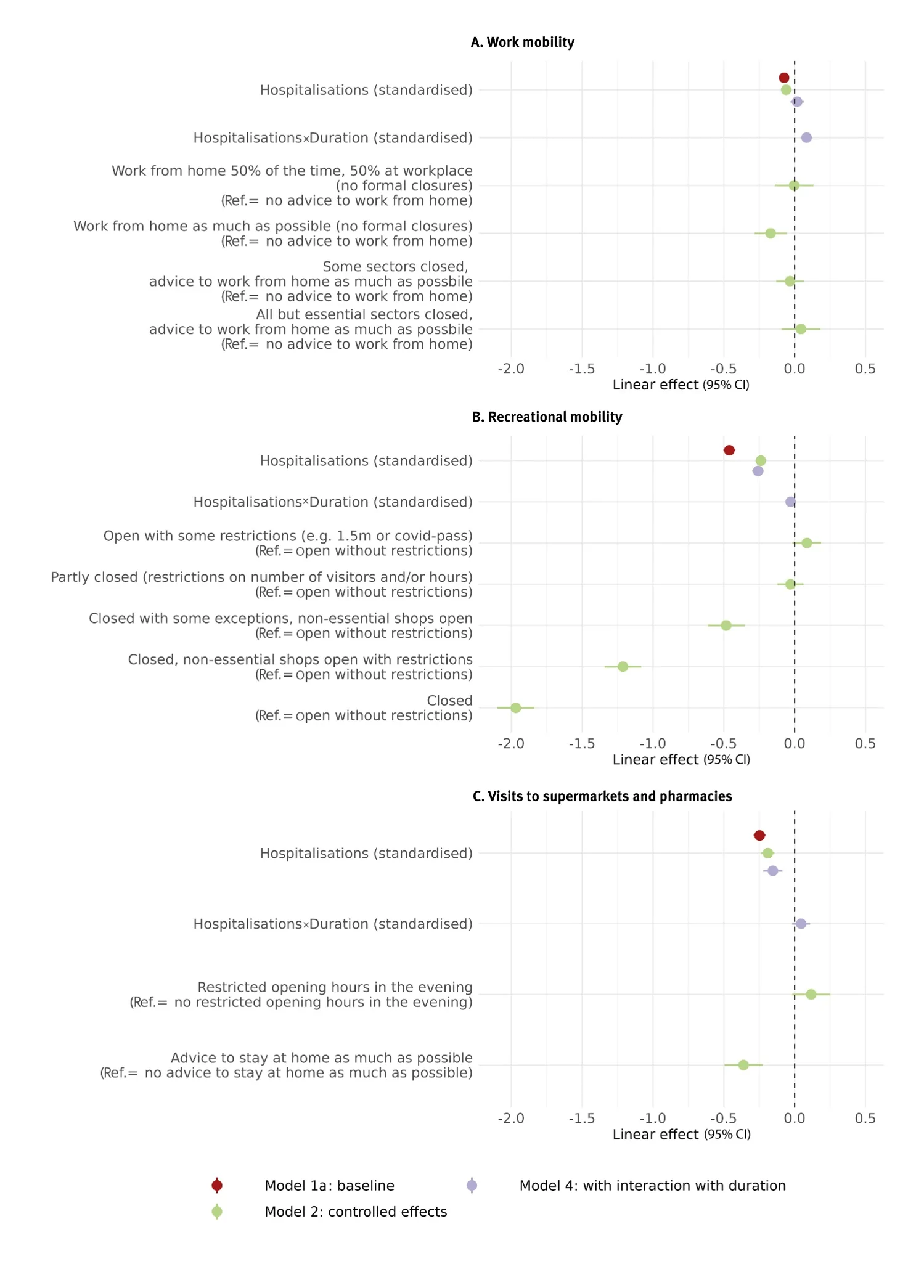
Time-series model parameters of pandemic severity and government response on behaviour among the population (mobility data), the Netherlands, 2020–2022 (n = 962 mobility data points)

CI: confidence interval; Ref: reference category; Interaction effects are estimated using multiplication (hospitalisations × duration).

For the impact of government response (providing behavioural advice and implementing restrictive policies, Hypotheses 1b and 1c), the findings varied considerably between measures and behaviours. Model 2 (Figures 3 and 4, controlled effects) showed that some behavioural advice was associated with increased adherence, for example advice on testing (average marginal effects up to 0.25). In contrast, several other advice measures had a small, no, or even a negative association with the targeted behaviours, such as limits on household visitors or the advice to work from home.

A similar mixed pattern emerged for restrictive policies. The controlled coefficients in Figure 4, Model 2 show that the clearest impact was found for recreational mobility: (partial) closures of these settings lead to very large reductions in mobility (linear effect up to −2). However, sector closures were not associated with reductions in work mobility, and limits on the opening hours of essential shops did not lead to fewer visits to these places. For the behaviours measured using surveys, only restrictive measures for elderly (age group not specified in the policy) and vulnerable people, closing care facilities and curfew were enforced measures. The coefficients in Figure 3, Model 2 show that these had an inconsistent impact on visiting and distancing behaviour in the entire group of participants (both positive and negative associations).

Regarding Hypothesis 2, Figure 3 shows that almost all associations between government response and protective behaviour were smaller when including hospitalisations in the models (Model 2), compared with the models that only included government response (Model 1b). Particularly for testing behaviour, the estimated impact of test advice became much smaller when accounting for self-guided behaviour change due to changes in pandemic severity (the coefficients in Model 1b reduced by 33% to 100% in Model 2). Although this comparison was not possible for the mobility behaviours, comparing Model 2 to Model 1a in Figure 4 shows that all estimates of the impact of pandemic severity became smaller when government response was included in the models. Hence, also in the mobility data, these variables partly explain the same variance in behaviour.

Regarding Hypothesis 3 on the signalling function of enforcement, results of Model 3 in Figure 3 indicate that more restrictive policies in general were not associated with more handwashing or testing behaviour and neither association was statistically significant. We also did not find consistent support for Hypothesis 4 on a reduction in impact of hospitalisations on behaviour over time. Model 4 in Figures 3 and 4 showed mixed patterns for the interaction between hospitalisations and duration.

### Sensitivity and exploratory analyses

The results of the sensitivity and exploratory analyses can be found in Supplement part 3. Most findings were robust against changes in the measures and models, but two differences stand out. Firstly, when removing holidays and changing the time lag between hospitalisations and behaviour, the estimated influence of advising testing at test facilities in Model 2 became negative (Sensitivity analyses 2a–c). This indicates that testing behaviour was not driven by the formal test advice, but rather by pandemic severity and availability of self-tests. Secondly, Sensitivity analysis 4, which measured testing behaviour by focussing on specific types of tests rather than all tests, showed mixed patterns, probably because people who performed other tests were coded as not having tested with these operationalisations. This highlights the importance of considering all types of tests to understand testing behaviour. Finally, our exploratory analyses indicate that mask mandates could have mixed effects on mobility: recreational mobility reduced when people were advised or required to wear a facemask, but the obligation to wear a mask was also associated with an increase in visits to essential shops; the specific data are appended in Supplement part 3, figures R.6.1 and R.6.2.

## Discussion

This study investigated the role of voluntary and enforced behaviour change during the Dutch COVID-19 pandemic. By studying how various forms of protective and risk behaviour were associated with changes in pandemic severity, behavioural advice and restrictive policies, we aimed to shed light on how the behaviour change necessary to mitigate infectious disease outbreaks can be achieved. Our results provide consistent evidence for self-guided behaviour change: increases in pandemic severity were associated with increases in all forms of protective behaviour except handwashing. The impact of behavioural advice and of restrictive policies was found to be inconsistent. (Partial) sector closures were for instance associated with reductions in recreational mobility, but not with other forms of mobility such as work-related travel. Advice to stay at home and work from home were associated with fewer visits to essential shops and workplaces, but advice on distancing from friends and family did not have a clear association with visiting friends and family or on keeping 1.5 m distance from them. So, although some forms of advice and restrictive measures were associated with more protective behaviour, other measures had no or even the opposite impact. Moreover, our results show that the impact of government advice and restrictive measures is overestimated when not considering self-guided behaviour change. We did not find support for a signalling function of restrictive policies, nor did we find consistent support for a reduction in the impact of pandemic severity on behaviour over time (risk habituation). This suggests that behaviour change could largely be attributed to voluntary responses to changing pandemic circumstances, even during later phases of the pandemic.

A key strength of this study is the use of two complementary longitudinal data sources, behavioural survey data and Google mobility data, which allowed us to capture a broad range of protective behaviours across different domains and time scales. Limitations include that we used single-country observational data, which limited causal inference and makes future, country-comparative research necessary to assess the generalisability of our results. Moreover, the restrictive measures for elderly (age group not specified in the policy) and vulnerable people were used to predict visiting and distancing behaviour in the whole sample. These results may thus underestimate the behavioural effects of these policies on this specific group. In addition, we proxied pandemic severity with hospitalisations, which may not fully capture how pandemic severity can shape people’s behaviour. Future research might use multi-country data and within–between study designs to further investigate the impact of different types of policies and self-guided protective behaviours. Moreover, by aiming to understand behaviour change, our approach did not consider the impact of this behaviour change on pandemic outcomes such as infections and hospitalisations. Future research might investigate to what extent observed levels of behaviour change are sufficient for pandemic mitigation.

The aim of this study was not to identify psychological, within-person predictors of behaviour change, nor to examine all the potential determinants of prevention behaviours during COVID-19. We sought to examine the relative influence of three contextual influences (pandemic severity, behavioural advice and restrictive policies) to better understand the impact of government responses. Considering our findings in relation to individual-level communication and behavioural research can help design effective mitigation strategies. Except for handwashing behaviour, all forms of behaviour varied considerably during the period under study, highlighting the potential for promoting protective behaviour through public health interventions. The consistent impact of pandemic severity shows that many people behaved responsibly and changed their behaviour when the risk of infection increased. Behavioural research indicates that this can be supported and strengthened through appropriate risk communication, clear instructions about what behaviour can help to protect against infections and why these behaviours are effective, and facilitating desired behaviour change [4,6,18]. Restrictive policies can also be effective in enforcing behaviour change, as shown for recreational mobility in our analyses, but it should be noted that restrictions or closures do not appear to always be effective in changing behaviour. Moreover, research shows that restrictive policies may have a negative impact on social trust [19,20]. It is therefore important to promote voluntary behaviour change through effective risk communication, practical behavioural advice, support and facilitation, and the selective application of restrictive policies accompanied by efforts to maintain social trust.

## Conclusion

Our study illustrates the feasibility and relevance of evaluating public health policy by studying change in the specific behaviours targeted to achieve public health outcomes. These results underscore the importance of distinguishing between voluntary and enforced behaviour change in studies evaluating the impact of restrictive policies. When voluntary behaviour change is not explicitly modelled, self-guided behaviour change could be misattributed as resulting from government enforcement.

## Data Availability

Population-level data and analysis codes are available on OSF (https://osf.io/7d3mn/overview).

## Supplementary Data

6933000 Supplementary Material
