# Supplementary material for: Disentangling enforced and voluntary behaviour change during the COVID-19 Pandemic, the Netherlands, 2020 to 2023
Source: Euro Surveill. 2026 Nov 5;31(44):2600078. doi: 10.2807/1560-7917.ES.2026.31.44.2600078 (PMC13743897; doi:10.2807/1560-7917.ES.2026.31.44.2600078)

Supplementary Material part 1 – Description of data and measures

This supplementary material is hosted by Eurosurveillance as supporting information alongside the article "Disentangling enforced and voluntary behaviour change during the COVID-19 pandemic in the Netherlands, 2020 to 2023", on behalf of the authors, who remain responsible for the accuracy and appropriateness of the content. The same standards for ethics, copyright, attributions and permissions as for the article apply. Supplements are not edited by Eurosurveillance and the journal is not responsible for the maintenance of any links or email addresses provided therein."

Datasets

In this study, we used data on protective behaviour among the Dutch Public, the Dutch governments' response and pandemic severity. These data were collected from different sources.

Our choice of behaviours was guided by the following principles:

1. *Number of observations in different pandemic situations*
   Studying the impact of government response and pandemic severity on behaviour over time requires longitudinal data on changes in the public's behaviour. Because government response and pandemic severity often coincided, the timespan of data collection should include multiple observations of behaviour under different combinations of these factors.
2. *Reliability of the data*
   Whenever possible, we used objective data of changes in behaviour. If no such objective data were available, we used self-reported behaviour data from the RIVM surveys that were shown to be valid and reliable (Bussemakers, van Dijk, Dima, & de Bruin, 2023).
3. *Scope of behaviours*
   Since our hypotheses are not specific to specific forms of protective behaviour and to ensure our findings could be relevant for future pandemic preparedness (which may require different protective behaviours), we aimed to include a diverse set of behaviours.

Table 1 provides an overview of behaviours and data sources that were identified and met these requirements while Table 2 includes the sources for the contextual data related to pandemic severity and government response.

The survey data included both a repeated cross-sectional trend study ('Trend study') and a dynamic cohort study ('Cohort study). A general description of the survey data collection and results can be found at https://www.rivm.nl/en/coronavirus-Covid-19/research/behaviour. More detailed information on the Cohort study has also been published in a Cohort Profile (Boom et al., 2023).

Table 1: Behaviours included in the study and their sources

Behaviour	Data source
Visits to retail and recreational facilities	Google Mobility database NL<br>retrieved February 2024 from https://www.google.com/covid19/mobility/
Working from home	Google Mobility database NL<br>retrieved February 2024 from https://www.google.com/covid19/mobility/
Visits to supermarkets	Google Mobility database NL

and pharmacies	retrieved February 2024 from https://www.google.com/covid19/mobility/
Handwashing Behaviour	RIVM Corona Behavioural Unit Covid-19 Cohort and Trend studies
Testing behaviour	RIVM Corona Behavioural Unit Covid-19 Cohort and Trend studies
Visiting behaviour	RIVM Corona Behavioural Unit Covid-19 Cohort and Trend studies
Keeping 1.5m distance from friends and family	RIVM Corona Behavioural Unit Covid-19 Cohort and Trend studies

Table 2 Contextual (pandemic) characteristics in the study and their sources

**Independent variables**	**Data source**
Government advice and policies	Covid-19 Measures Timeline (National Institute Public Health and Environment). Retrieved Jan-June 2024 from https://www.rivm.nl/gedragsonderzoek/tijdlijn-van-coronamaatregelen-2020
	Complete Overview Pandemic Reconstruction (Dutch Safety Board). Retrieved Jan-June 2024 from https://coronaonderzoek.onderzoeksraad.nl/totalereconstructie
	Timeline: Measures and Press Conferences (Dutch Ministry Public Health, Welfare and Sport). Retrieved Jan-April 2024 from https://www.eerstekamer.nl/overig/20230914/interactieve_tijdlijn_maatregelen_4/document
	COVID-19 Government Response Tracker (Blavatnik School of Government, University of Oxford). Retrieved April 2024 from https://github.com/OxCGRT/covid-policy-dataset
Covid-19 hospitalizations	Covid-19 hospitalisations (National Institute Public Health and Environment). Retrieved April 2024 from https://data.rivm.nl/meta/srv/dut/catalog.search#/metadata/4f4ad069-8f24-4fe8-b2a7-533ef27a899f
School and official holidays	Official Holidays (National Government) Retrieved May 2024 from https://www.rijksoverheid.nl/onderwerpen/schoolvakanties/vraag-en-antwoord/officiele-feestdagen
	Holidays and Vacations 2020-2022 (Ondernemen met Personeel). Retrieved May 2024 from https://www.ondernemenmetpersoneel.nl/feestdagen-en-schoolvakanties-2022

Measures

Self-reported survey data and related control variables

Handwashing, testing and visiting behaviour were measured using survey data. Handwashing was measured by asking people “In the past 7 days (1 week), approximately how often per day did you wash your hands with soap and water?”. The following answer options were provided: 0, 1-2 times, 3-5 times, 6-10 times, 11-15 times, 16-20 times and >20 times. These categories were recoded to their midpoints (0, 1.5, 4, 8, 13, 18 and 23)

Visiting behaviour was measured using the question how often people received friends or family in their own home (not including visits for informal care) or visited friends or family in the past week. We created a dichotomous variable (yes/no) indicating whether participants avoided visits with friends and family in the past week.

Keeping 1.5m distance from friends and family was asked to people who either visited friends and family or received visits from friends or family in their own home at least once during the past week. The question asked them how often these people came closer than 1.5m distance from them during the last visit. This question originally had six categories (ranging from never to very often), but an earlier analysis indicated that similar categories need to be merged to form a reliable measure (Bussemakers et al., 2022). We therefore created a variable measuring distancing when visiting with friends and family with three categories:

1. Yes (people who did not visit with friends or family or who indicated that during the last visits, friends and family were never within 1.5m from them)
2. No, infrequently closer than 1.5m from others (original answers: others seldom/sometimes closer than 1.5m)
3. No, frequently closer than 1.5m from others (original answers: others regularly/often/very often/always closer than 1.5m)

When analysing trends in distancing over time, it became clear that the most notable change was found among people who violated this advice frequently, while the share of people who did so infrequently was relatively stable. This could be because when people adhere to distancing guidelines more, people who initially did so frequently move into the ‘infrequently’ group, while the people who did so ‘infrequently’ moved into the reference group who was seldom or never within 1.5m from others. In other words, it is unclear how changes in adherence translate into changes in the share of people who distance infrequently, so we decided to focus on changes in ‘frequently’ in our analyses (relative to the people who did keep distance).

It should also be noted that the measure differs slightly between surveys. Both the Trend survey and the first 7 rounds of the Cohort survey included separate questions on keeping 1.5m distance when participants <u>visited</u> friends or family vs. when participants <u>received</u> friends or family at their own home, while the Cohort surveys from round 8 onwards included one question for distancing during the last visit (regardless of whether this was at their own home or the home of their friends or family). Since participants who answered both questions reported very similar distancing behaviour in both situations (kappa >0.5), we combined their answers and they received their highest score on the behaviour measure (not their average score because of the categorical nature of the variables).

Testing with symptoms was measured by asking people whether they had experienced any Covid-related symptoms in the past 3 or 4 weeks (Trend Study) or 6 weeks (Cohort Study) and if so, whether they got tested and what type of test they had done. Based on this variable, we created a dichotomous variable indicating for the people who had symptoms, whether they tested or not (yes/no). People without covid-related symptoms or who indicated the symptoms were (likely or certainly) due to underlying health conditions have a missing value and are not included in the analyses of that specific wave, as these people do not have relevant testing behaviour. Question 28 provides more information on robustness analyses using alternative operationalisations of this variable.

Because differences in the sample composition of the Trend and Cohort study could affect average levels of protective behaviour, we used the following categorical control variables to estimate trends

in behaviour over time: Sex (Male, Female, Other), Age (5 categories, ranging from 16-24 to 70+), Education (Low, Middle and High) and Employment (yes/no).

Mobility behaviour (Google mobility data) and related control variables

Visits to retail and recreational facilities, working from home and visits to supermarkets and pharmacies were measured using Google Mobility reports. These data represent percentual changes in the number of visits to, respectively, retail and recreational facilities (shops and malls [not including supermarkets and pharmacies], bars, restaurants and cultural facilities), workplaces, and supermarkets/pharmacies. Data are reported daily and measured relatively to the pre-Covid period January 3-February 6 2020.

Because data are reported on a daily basis, it is important to control for weekly and seasonal fluctuations in these mobility patterns. Specifically, we control for day of week (categorical variable with 7 categories), summer school holidays (dichotomous, yes/no) and other school holidays (dichotomous variable, yes/no), official holidays (dichotomous variable, yes/no). Additionally, when investigating changes in working from home, we also control for whether school closures were in place, as these can force parents to work from home (categorical variable with 3 categories: no closure, closures at some educational types/levels and complete closures).

Contextual variables

For each behaviour, we included a categorical variable indicating the type of behavioural advice or restrictive policies the Dutch government provided at a time. Categories are specific for each behaviour:

- Working from home: this variable is a combination of the extent to which the Dutch government advised people to work from home and whether there were formal workplace closures.
- Retail and recreational sector: this variable indicates whether these sectors were closed or open and whether restrictions limiting the number of visits were in place when they were open (such as maximum number of visitors or requirements to ensure 1.5m distance). The Dutch government never advised not to visit these sectors when they were open, so for this behaviour we only have restrictive policies, no behavioural advice. Note that for the retail sector, we focus on closures of non-essential shops, as visits to essential shops (supermarkets and pharmacies) are analysed separately.
- Supermarkets and pharmacies: the Dutch government never advised people avoid supermarkets and pharmacies, but there have been some restrictions on their opening hours in the evening. Moreover, there were periods when people were advised to ‘stay home as much as possible’ which could have led to a reduction in people’s visits to supermarkets and pharmacies. Both are therefore included in the government response for this measure.
- Handwashing: at the start of the pandemic, the Dutch government introduced ‘basic behaviour rules’ which included handwashing guidelines. Although these ‘rules’ were never enforced, they were only formally scaled down to ‘advice’ in March 2022. We will regard both options as behavioural advice but will study whether adherence changes due to this change in government approach.

- Visiting friend and family: this variable indicates whether formal restrictions were in place that limited visits to vulnerable and elderly people (the closure of elderly care facilities for visitors) and the governments' advice for the entire public to limit visitors to a certain number (e.g., max 2 per day). The period during which it was advised to receive max. 1 visitor per day coincided fully with the implementation of a national curfew, so these variables were combined. Since this period also fully coincided with formal closures of non-essential workplaces, the retail and recreational sector and reduced opening hours of essential shops, this curfew was only relevant for visiting behaviour and not for the other behaviours under study.
- Keeping 1.5m distance from friends and family: this variable indicates whether the Dutch government advised to keep 1.5m distance from others. Since it is easier to keep 1.5m distance from others when fewer people are present, we also include the measure on limiting the number of visitors (see visiting friend and family above).
- Testing when experiencing symptoms: this variable includes the type of tests that were available for people who experienced Covid-19 related symptoms as well as the type of test that was advised for people with such symptoms.

Additionally, we measured general enforcement using a modified version of the Oxford Government Response Tracker (also called 'stringency index'). We calculated an overall enforcement score based on whether there were partial or full closures in schools, workplaces, public events, as well as narrow or extensive restrictions on gatherings, visits to elderly and vulnerable people and required use of face masks. The variable does not consider enforced stay at home orders, closures of public transport and restrictions on internal movement, as these have not been part of the Dutch governments' pandemic response. Moreover, unlike the original index, our modified index does not consider behaviour advise and recommendations, as we already measure these with our behaviour-specific advice variables; the index is only used to investigate the signalling function of enforcement.

In the Netherlands, pandemic communication and response were mainly based on changes in the number of infections, hospitalisations and ICU-admittances of Covid-19 patients as indicators of pandemic severity. Of these measures, hospitalisations provides the most reliable indicator of pandemic severity as communicated to the Dutch public through government and media channels. The measure is available throughout the entire pandemic and reflects pandemic severity because it indicates how many people become seriously ill due to Covid-19. This means that when population immunity increases due to for instance vaccination campaigns, the resulting reduction in pandemic threat would translate in fewer hospitalisations and thus a lower score on the pandemic severity indicator.[1] To measure hospitalisations, we used the average number of hospitalisations in the week behaviour was measured. In this way, we account for the fact that there could be small day-to-day fluctuations in hospitalisations and registrations, as well as for the fact that there will be a short time-lag between changes in hospitalisations and the moment communication about these changes will affect people's behaviour. For testing behaviour, we use the average number of hospitalisations in the previous 3, 4 or 6 weeks, depending on the period the survey question referred to.

[1] The other indicators are less useful for our study. For the number of infections, there is no indicator available throughout the entire pandemic (e.g., number of positive tests is only available when large-scale testing was implemented and before the introduction of self-tests) and it also does not adequately reflect that the health consequences of Covid-19 changed over time. For ICU-admittances, it can take relatively long for increases in pandemic threat to be visible in increases in admittances, while the measure is also affected by changes in hospital policies and treatment options.

Finally, pandemic duration was calculated as the number of days since the start of the pandemic in the Netherlands (as indicated by the first confirmed case on 27 Feb. 2020).

<u>Missing data</u>

At the contextual level, the measure for general enforcement policies based on the Government Response Tracker (stringency index) was only available until December 2022, while we use survey data for 2023 as well. However, since the last enforced behavioural policies ended in May 2022 in the Netherlands, we code this variable as 0 (no enforcement policies) after that date.

The first round of the Trend Study (conducted on 28 September 2020) did not include a measure for employment and could therefore not be included in the analyses. The loss of information is small, because the Cohort study was also conducted around the same time (30 September 2020).

Moreover, some participants in the surveys also had missing data. About 10% of observations had missing data on the behaviour questions, mostly because not all participants of the Cohort Study were asked about their behaviour the first time they participated (Boom et al., 2023). Because this concerns the dependent variable, participants with missing data on a behaviour are excluded from the analyses of that behaviour. Moreover, less than 1% of observations had missing data on the control variables (0.07% on sex, 0% [6 observations] on age, 0.96% on education, and 0.39% on employment) . Because this group is very small, they were also excluded from the analyses.

Supplementary Material part 2 – Time series models with posterior analyses

This supplementary material is hosted by Eurosurveillance as supporting information alongside the article "Disentangling Enforced and Voluntary Behaviour Change During the COVID-19 Pandemic in the Netherlands", on behalf of the authors, who remain responsible for the accuracy and appropriateness of the content. The same standards for ethics, copyright, attributions and permissions as for the article apply. Supplements are not edited by Eurosurveillance and the journal is not responsible for the maintenance of any links or email addresses provided therein."

Model description

Each model was based on two regression equations, one for changes in mobility and one for changes in pandemic severity measured by hospitalizations. Below are the equations for Model 4, the full model that includes the linear interaction between hospitalisations and duration.

-
- $Y_{mob,t} = \gamma_o + \alpha \cdot Hosp_{t-1} + \alpha_{dur} \cdot Hosp_{t-1} \cdot Dur_t + \sum_{(i=1,\ldots,n)} \gamma_i \cdot Government\ Response_{(i,t)}$
- $\epsilon_{mob,t} \sim N(o, \sigma 2_{mob})$
-
- $Y_{hosp,t} = \beta_o + \beta \cdot Mob_{t-18} + \beta_{hosp\_aut} \cdot Hosp_{t-1} + \delta_{dur\_hosp} \cdot Dur_t + \epsilon_{hosp,t}$
- $\epsilon_{hosp,t} \sim N(o, \sigma 2_{hosp})$

Control variables capture the effects of day-of-week, official holidays, summer holidays, other school holidays as well as days preceding main holidays. When modelling work mobility, we also included school closures as a predictor. The inclusion of a variable indicating the days preceding specific holidays was not preregistered, but it was included because the descriptive analyses showed substantial changes in behaviour on these days as well.

Because we aimed to test hypotheses with H_0 of no association between the variables of interest and behaviour change, we used uninformed priors with a mean of 0 and variance of 20. Since we observed particularly large drops in mobility during holidays, we used a variance of 50 for the holiday control variables. Small effects were expected for duration and the feedback loop from behaviour on hospitalisations, so we set the variance of these coefficients to 5.

We used a lag of 18 days in the feedback loop from behaviour to hospitalisations[2]. As a result, the first 19 days for which mobility data was observed are not included in the model. One specific work-related behavioural advice was only implemented during this period (working from home in Brabant, one of the twelve Dutch provinces, only) and was therefore not included in the analyses.

Software

The models were estimated using RStan.[3] All models were estimated using 4 chains of 100.000 iterations.

Posterior checks

---

[2] Klinkenberg, D., Backer, J., de Keizer, N., & Wallinga, J. (2024). Projecting COVID-19 intensive care admissions for policy advice, the Netherlands, February 2020 to January 2021. Eurosurveillance, 29(10), 2300336.

[3] Stan Development Team (2025). "RStan: the R interface to Stan." R package version 2.32.7, https://mc-stan.org/.

The RHat of each model and coefficient was 1 and the effective sample sized ranged between 60.422 and 343.285, indicating the models converged well. Trace plots for the main coefficients of the full models are displayed in Figure A1 below. They indicate that all chains mixed well, and parameters ranged within a reasonable interval. Density plots of the main coefficients in the full model are displayed in Figure A2 and indicate these parameters all had a unimodal distribution.

Naturally, the variables measuring pandemic severity and government response were correlated, since they co-occurred in specific time periods (see Figure 1 and 3 indicating descriptive trends). As part of the preregistration, we derived VIF-scores from regression models including all predictors to check whether the variables could be included in the models. Fortunately, pandemic severity and behaviour-specific government response could be included in the same models (VIF <5) and only general enforcement correlated too strongly with the other measures. As a result, hypothesis 3 could not be tested for the mobility outcomes (as indicated in the preregistration).

This correlation between the explanatory variables also resulted in moderate correlations in the estimated parameters. These are shown in Figure A3, again derived from the full model. The correlations between coefficients of different types of government response (gamma) were most profound, because these have a common reference category. Some of the levels of government response also correlated with alpha, which indicated the impact of pandemic severity. No correlations were found with beta, which indicated the feedback loop from behaviour on pandemic severity. This indicates the correlations in parameter estimates indeed resulted from the factual co-occurrence of pandemic severity and government response, which is a key-part of this study and not a result of model misspecification.

Predictions

To assess the validity of the models, we also compared the observed trends in mobility to the predicted trends based on the full models. These are displayed in figure A4 below.

Overall, the models capture the trends in both mobility and hospitalisation very well, supporting the validity of the models. However, two differences stand out that should be discussed.

First, the models for work mobility do not fully capture the drops in visits to workplaces that occurred during the summer holiday periods. This is because the models include one coefficient to capture behaviour change caused by the entire (school) holiday period, while mobility varies also within that period. Since these summer-holidays were periods with relatively low levels of hospitalisations and stable policies, it means the differences in mobility during periods with higher and lower levels of hospitalisations are somewhat underestimated by the model. Hence, the impact of hospitalisations on mobility might be somewhat larger than estimated in these models. In other words, the estimated impact of hospitalisations on mobility in our models is conservative. Since we find a substantial impact of hospitalisations on work mobility in all models, this does not impact our conclusions.

Second, there was an increase in visits to essential shops during the second Covid-wave in the Netherlands (one year in the pandemic, during winter 2021) which is not captured by the model. Instead, the model estimates stable levels of mobility because during this period, hospitalisations and measures were relatively stable as well. Although the model also includes the interaction term between duration and hospitalization, this cannot capture the increase in mobility over this short time-period. This does not affect our conclusions regarding the estimated effects of hospitalisations and measures for this type of mobility. For our hypotheses on risk habituation, it indicates that although we do not find a reduction in the impact of hospitalisations measured throughout the entire pandemic period, some short-term risk habituation or fatigue may have occurred within periods

with high levels of pandemic severity and strict policies. We did not formulate a hypothesis on such short-term risk habituation process, but this may be further explored in future research.

Figure A1: Traceplots of the full models

Figure A1.1 – Work mobility

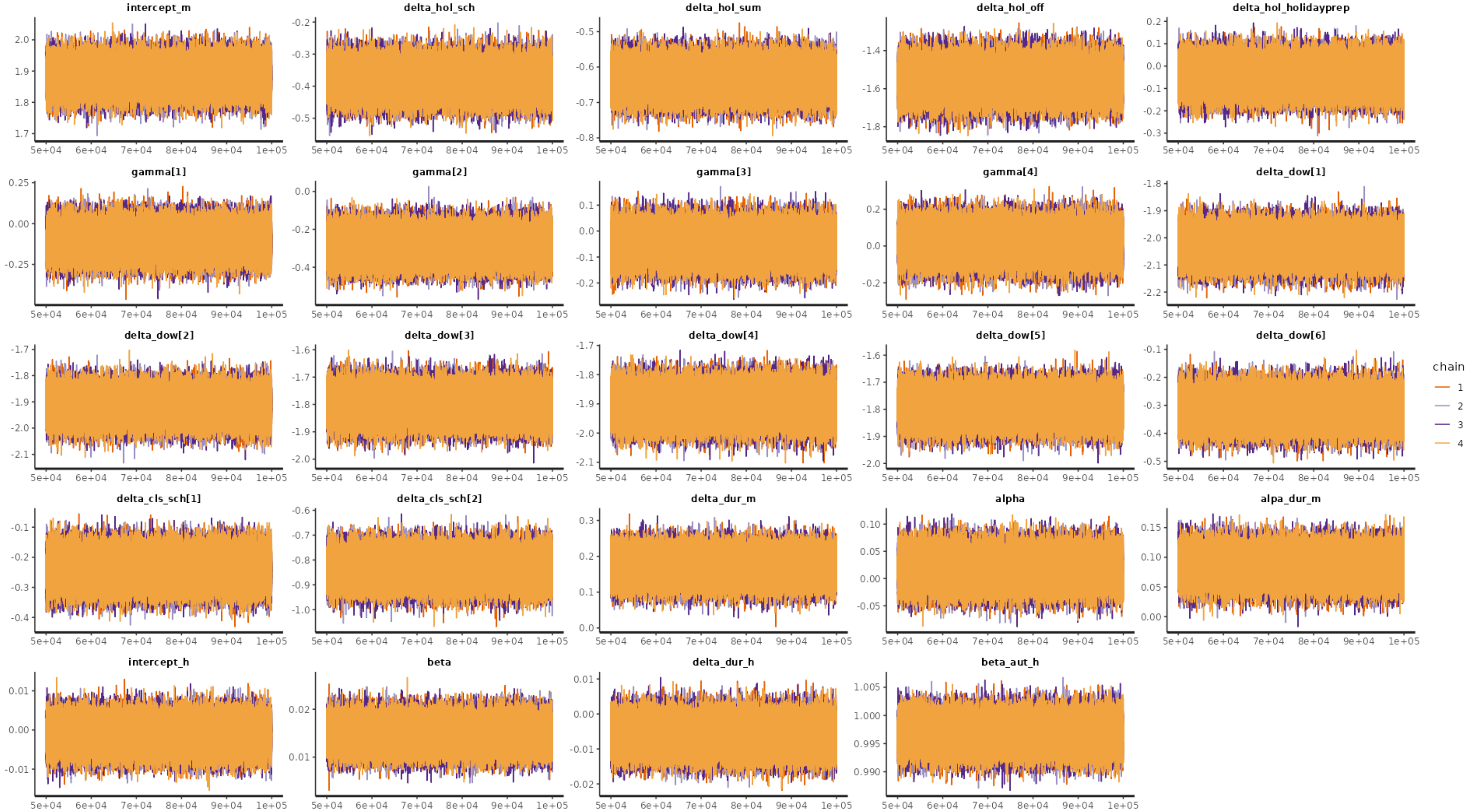

Figure A1.2 – Recreational mobility

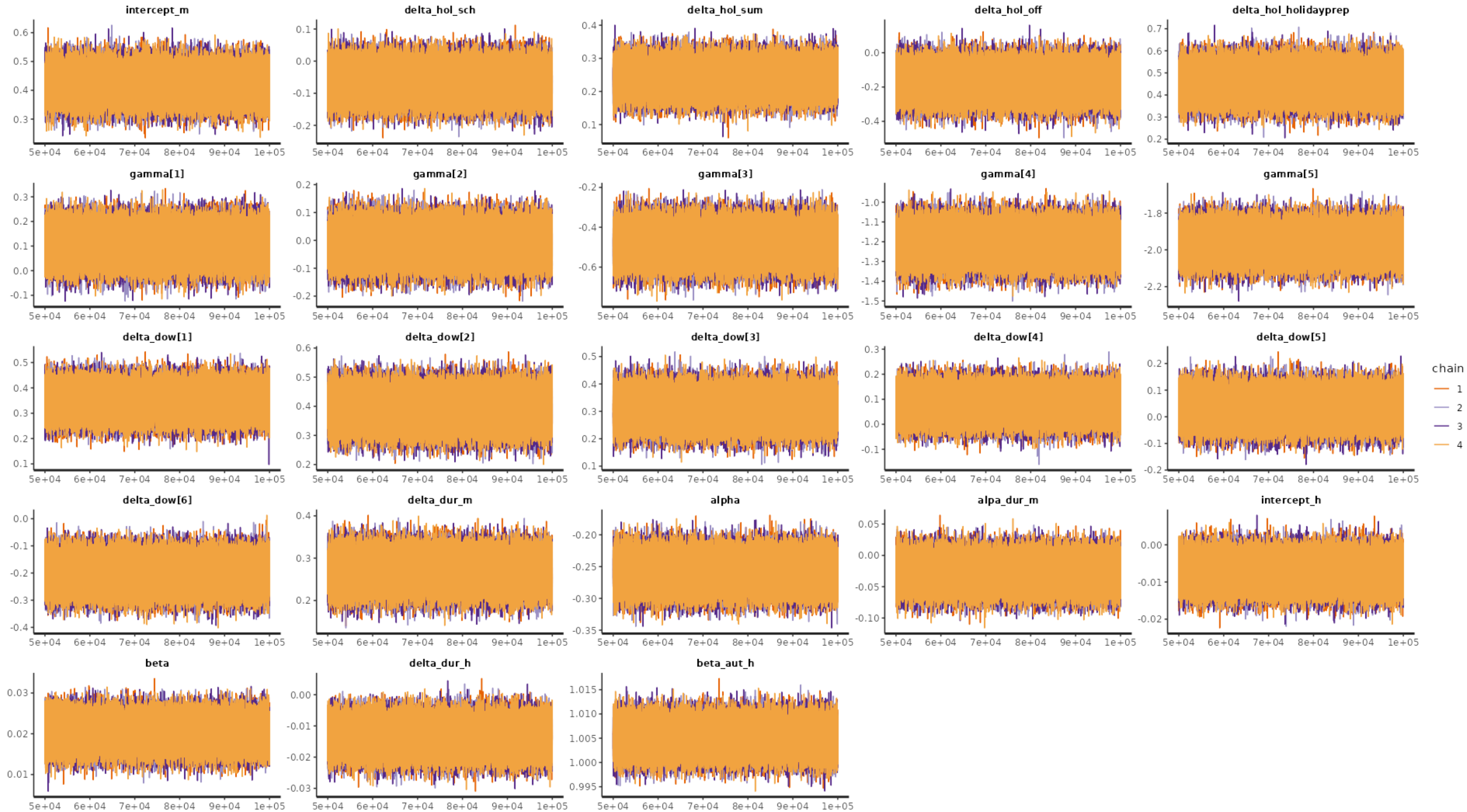

Figure A1.3 – Visits to essential shops

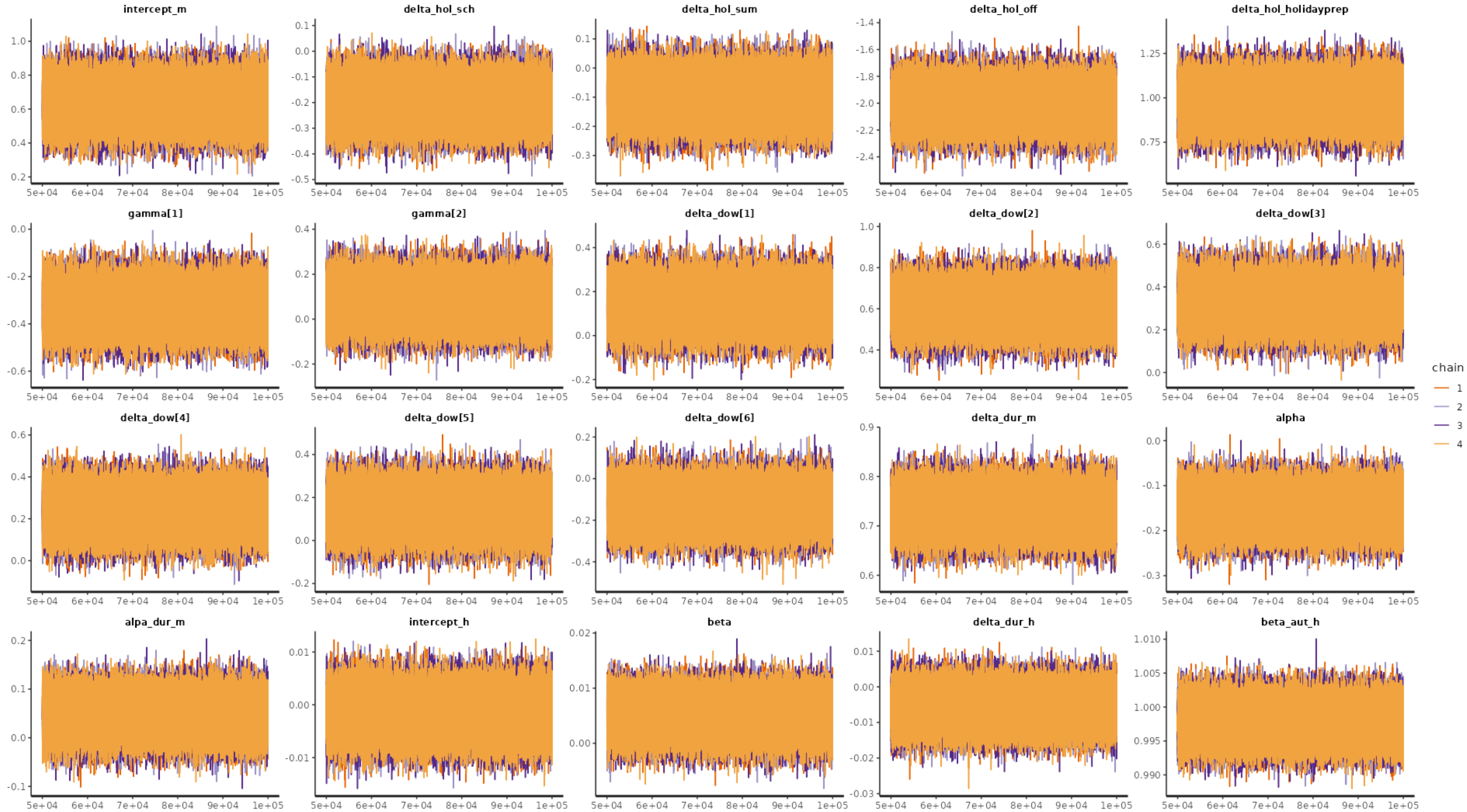

Figure A2: Density plots of the full models

Figure A2.1 – Work mobility

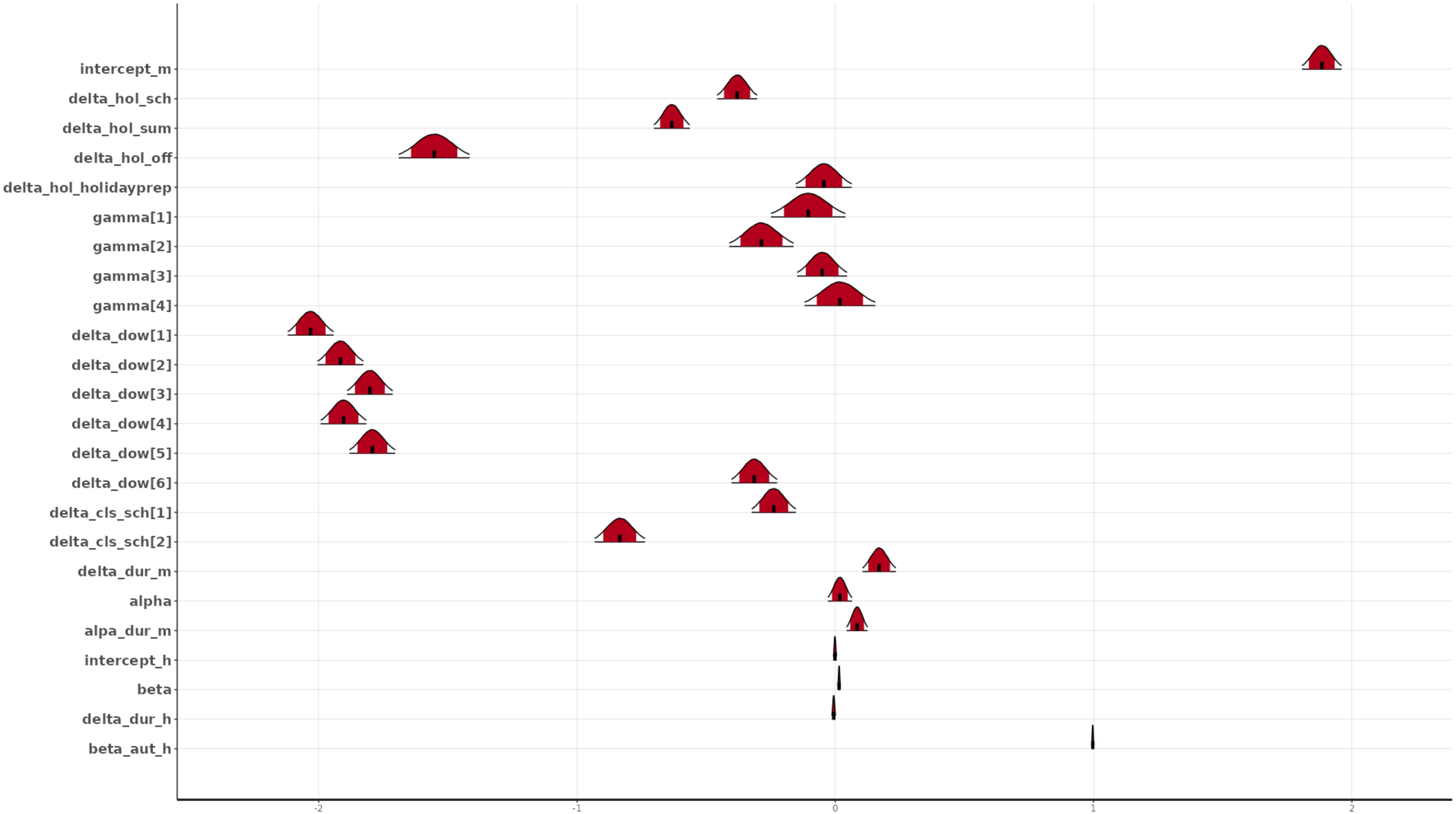

Figure A2.2 – Recreational mobility

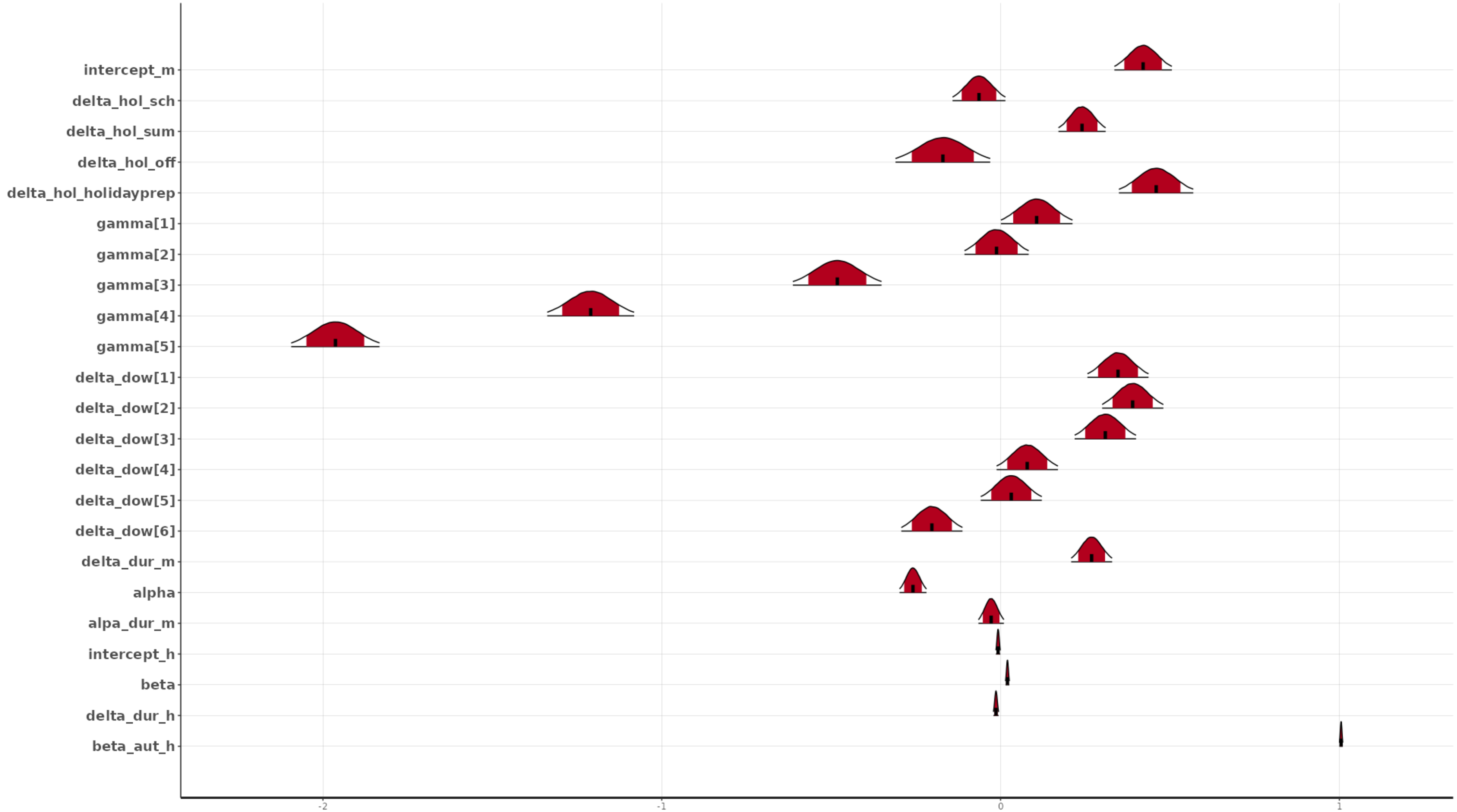

Figure A2.3 – Visits to essential shops

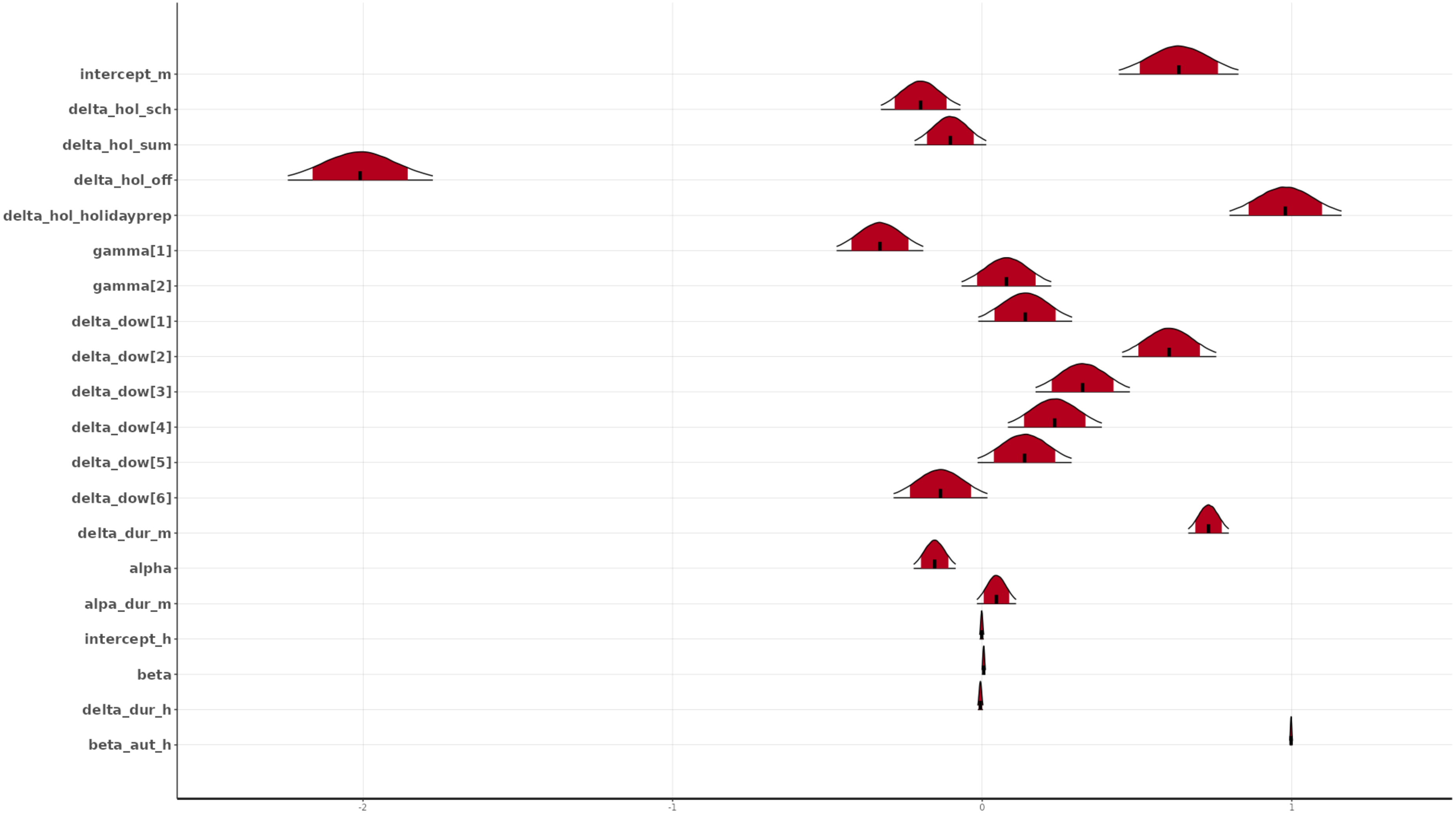

Figure A3: Density plots of the full models

Figure A3.1 – Work mobility

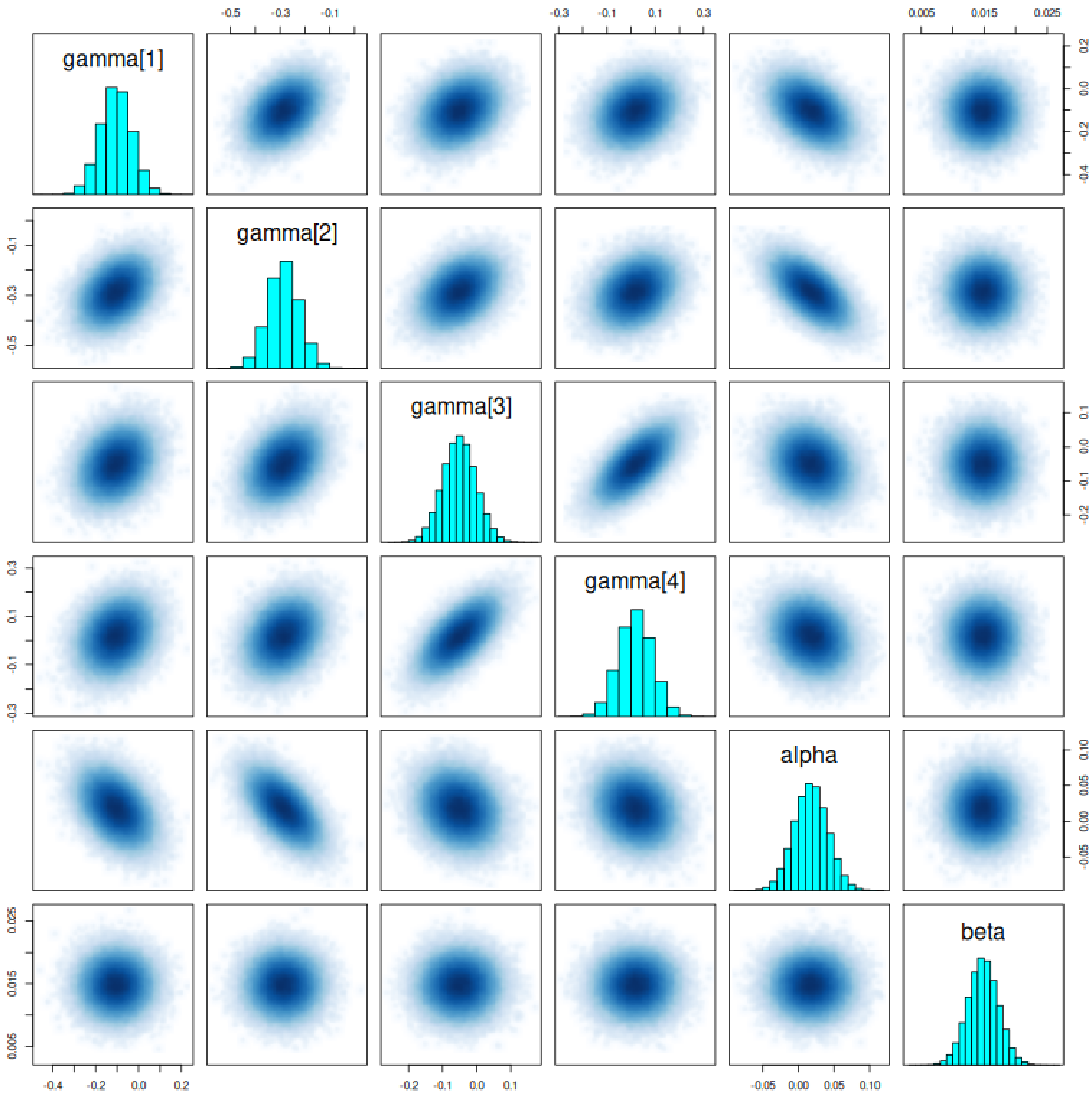

Figure A3.2 – recreational mobility

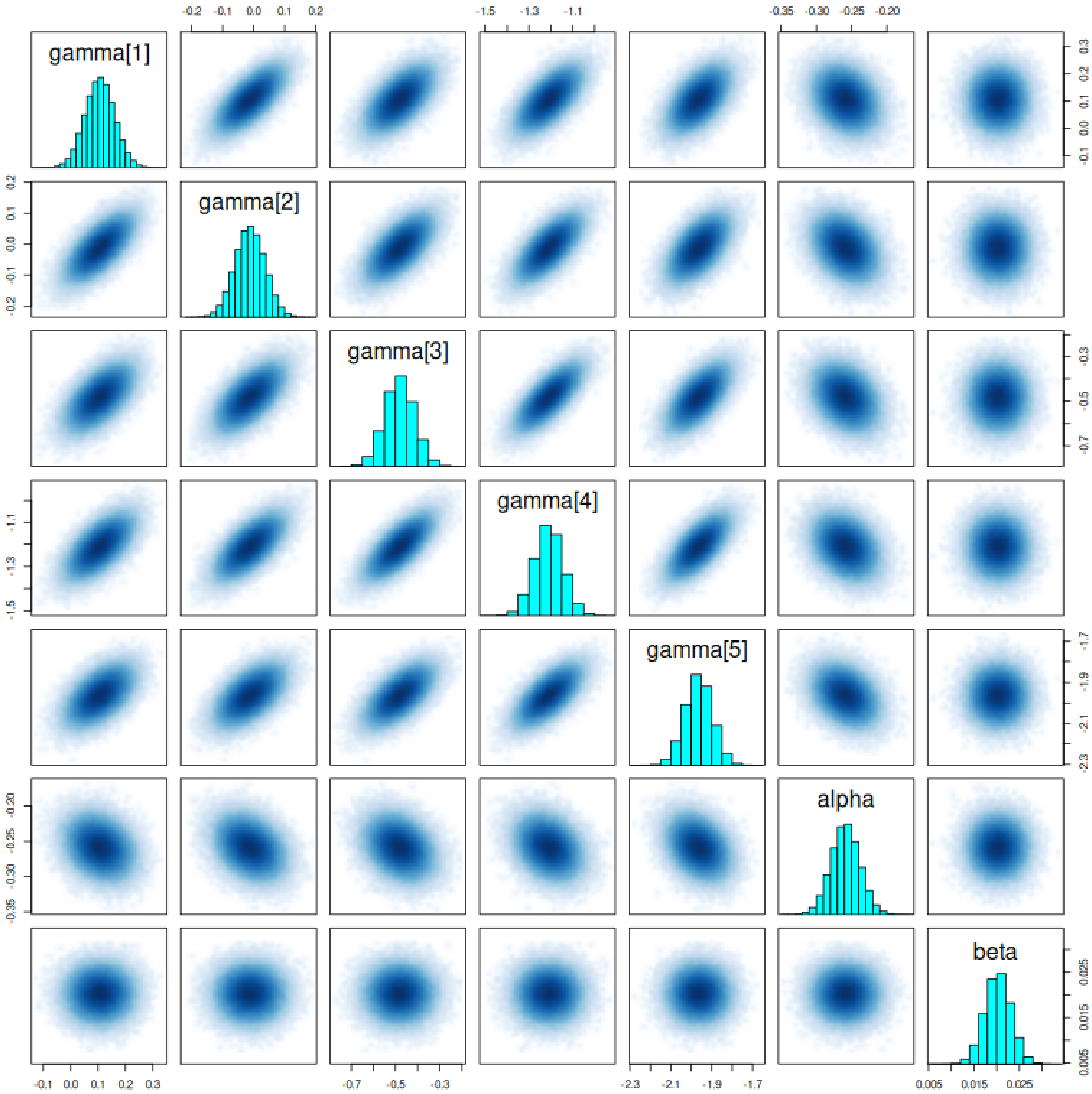

Figure A3.3 – Visits to essential shops

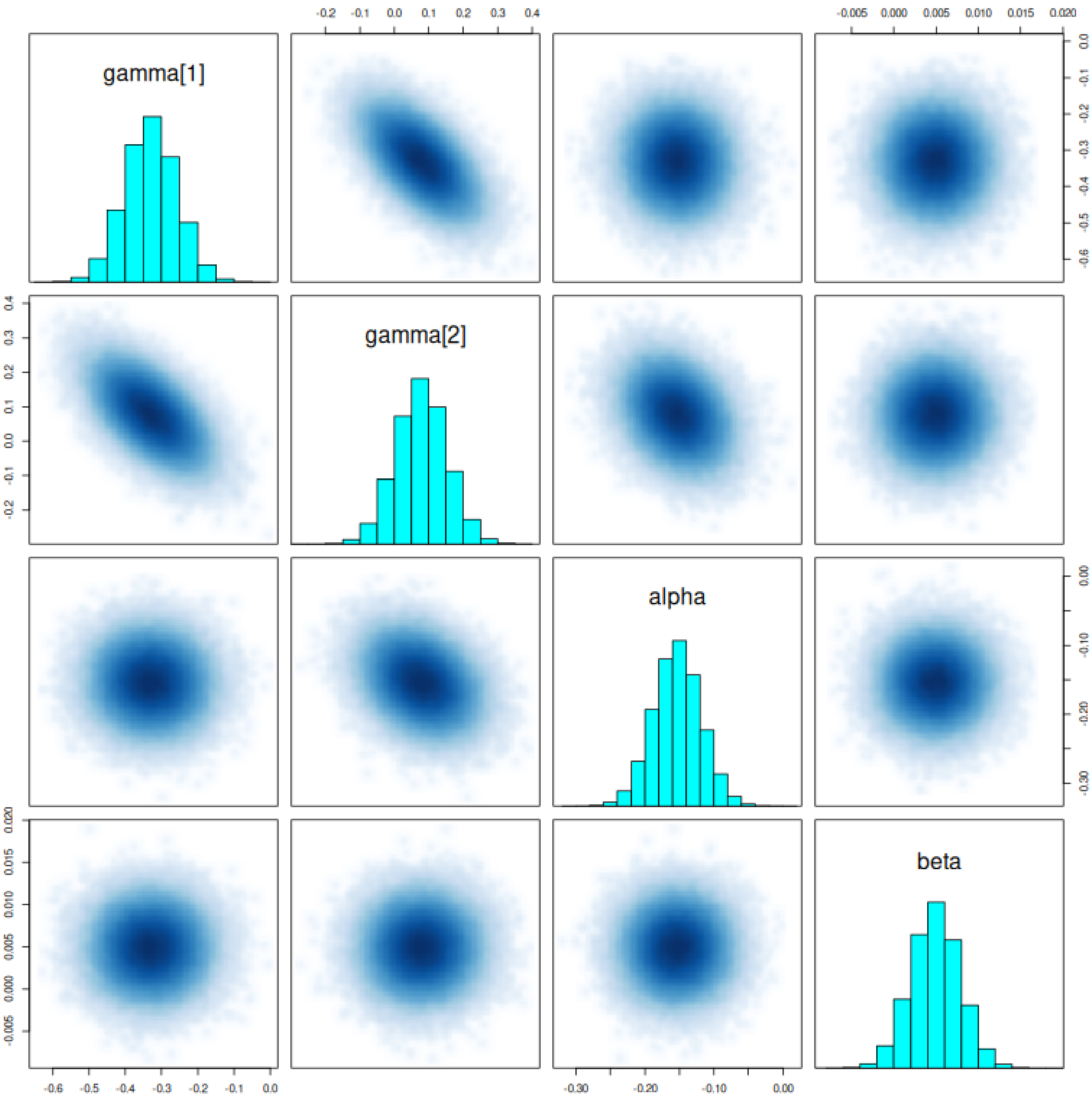

Figure A4: Observed and predicted trends based on the full models

Figure A4.1 – Work mobility

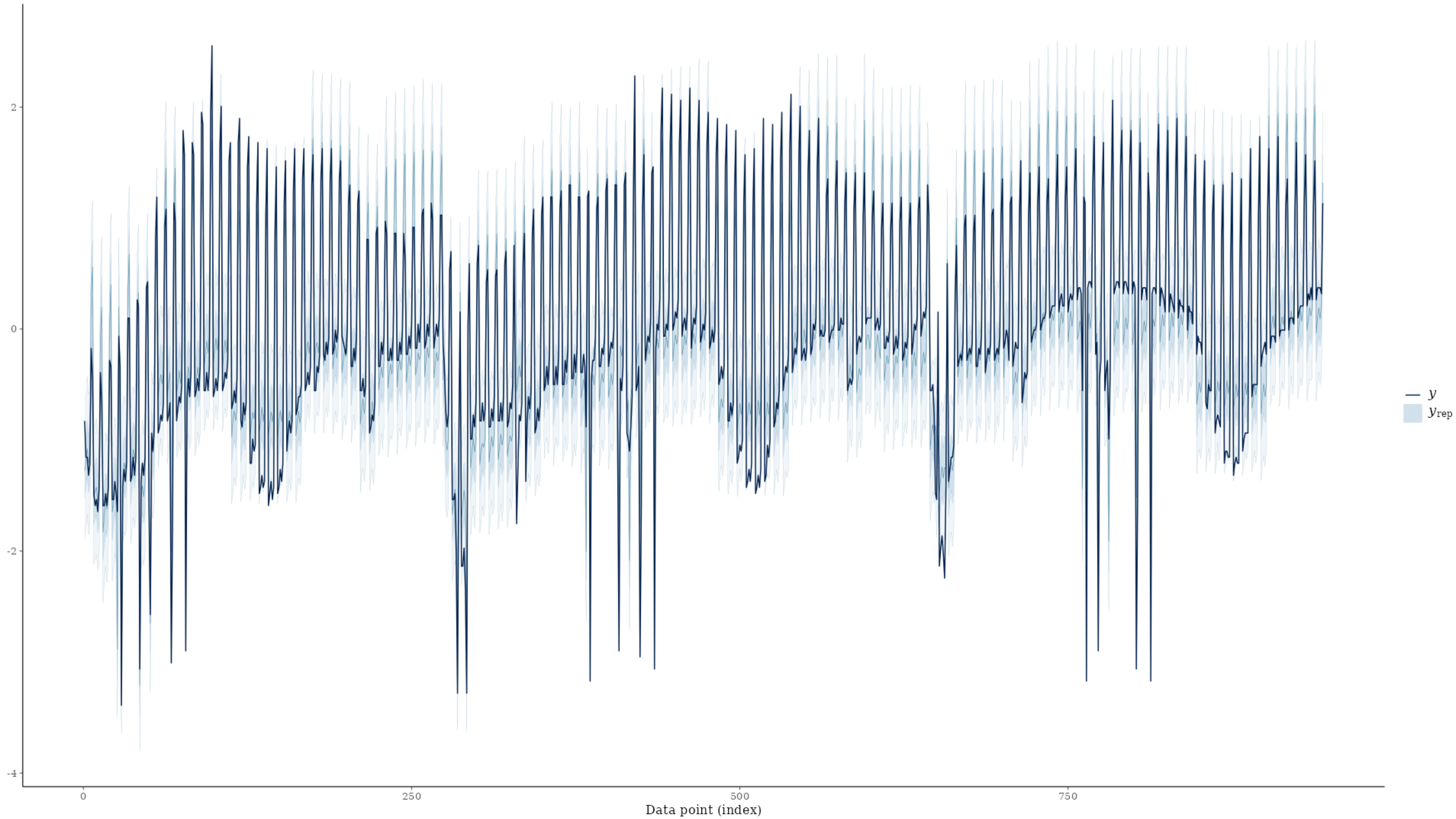

Figure A4.2 – Hospitalisations (Work mobility model)

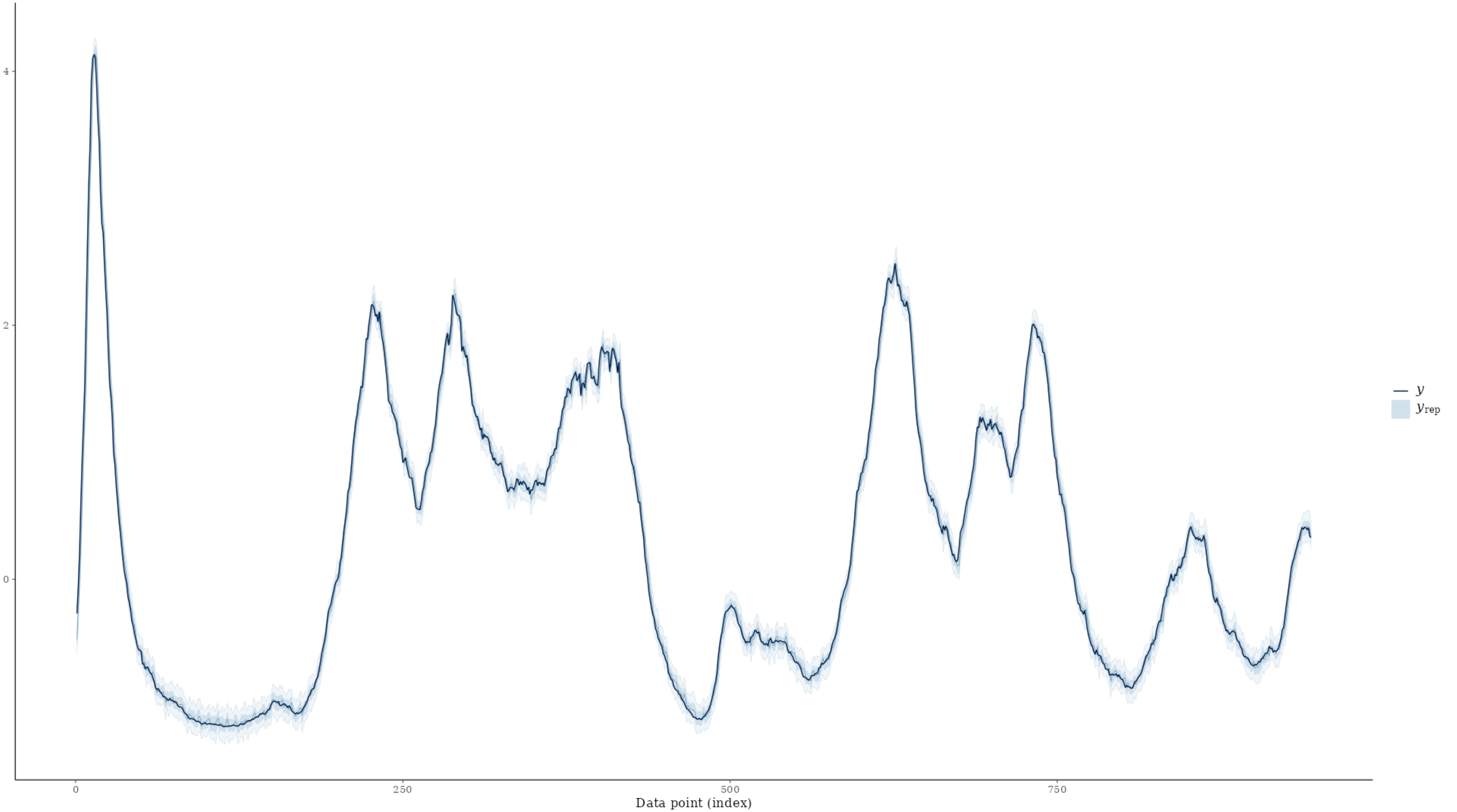

Figure A4.3 –Recreational mobility

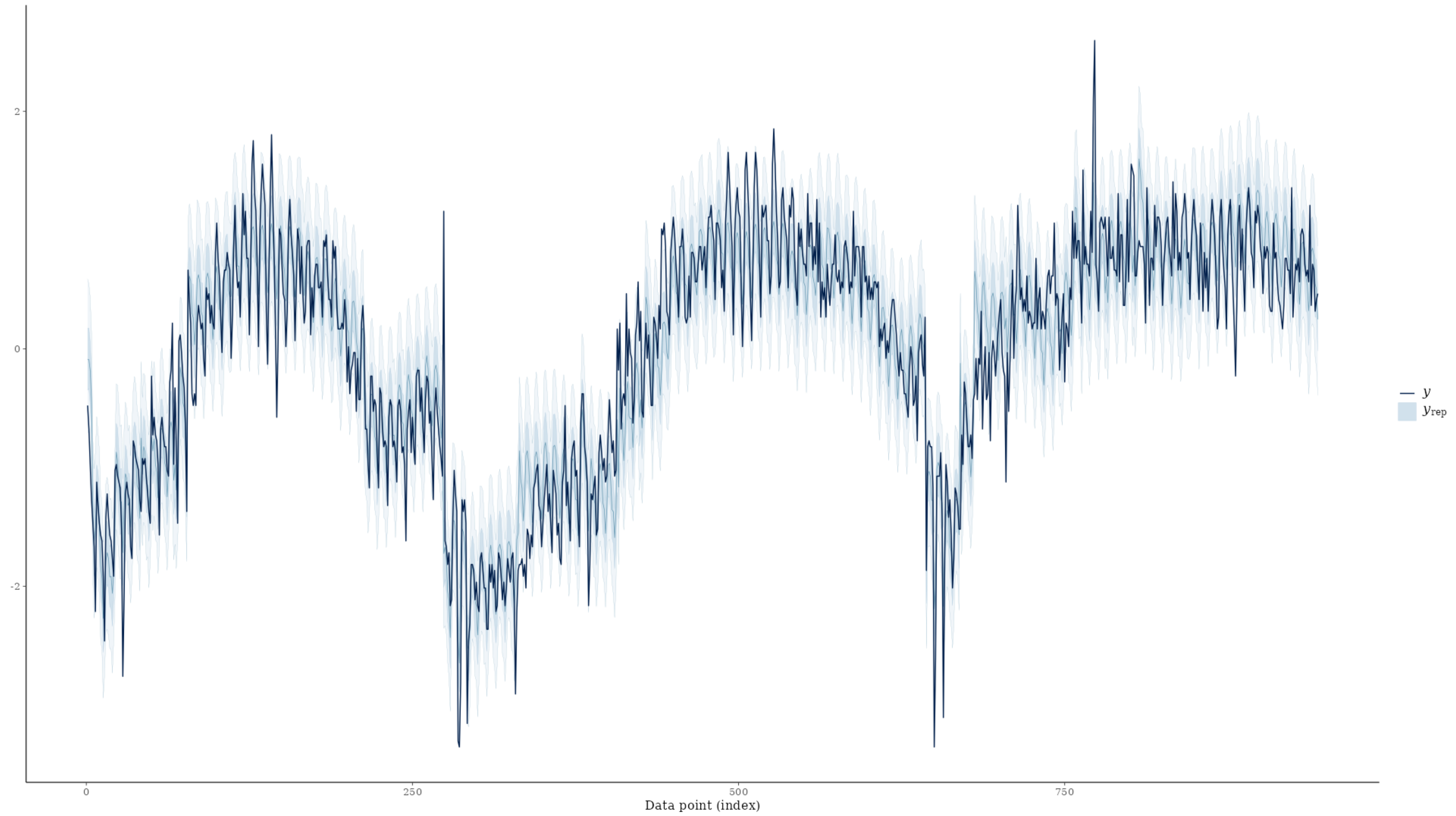

Figure A4.4 – Hospitalisations (Recreational mobility model)

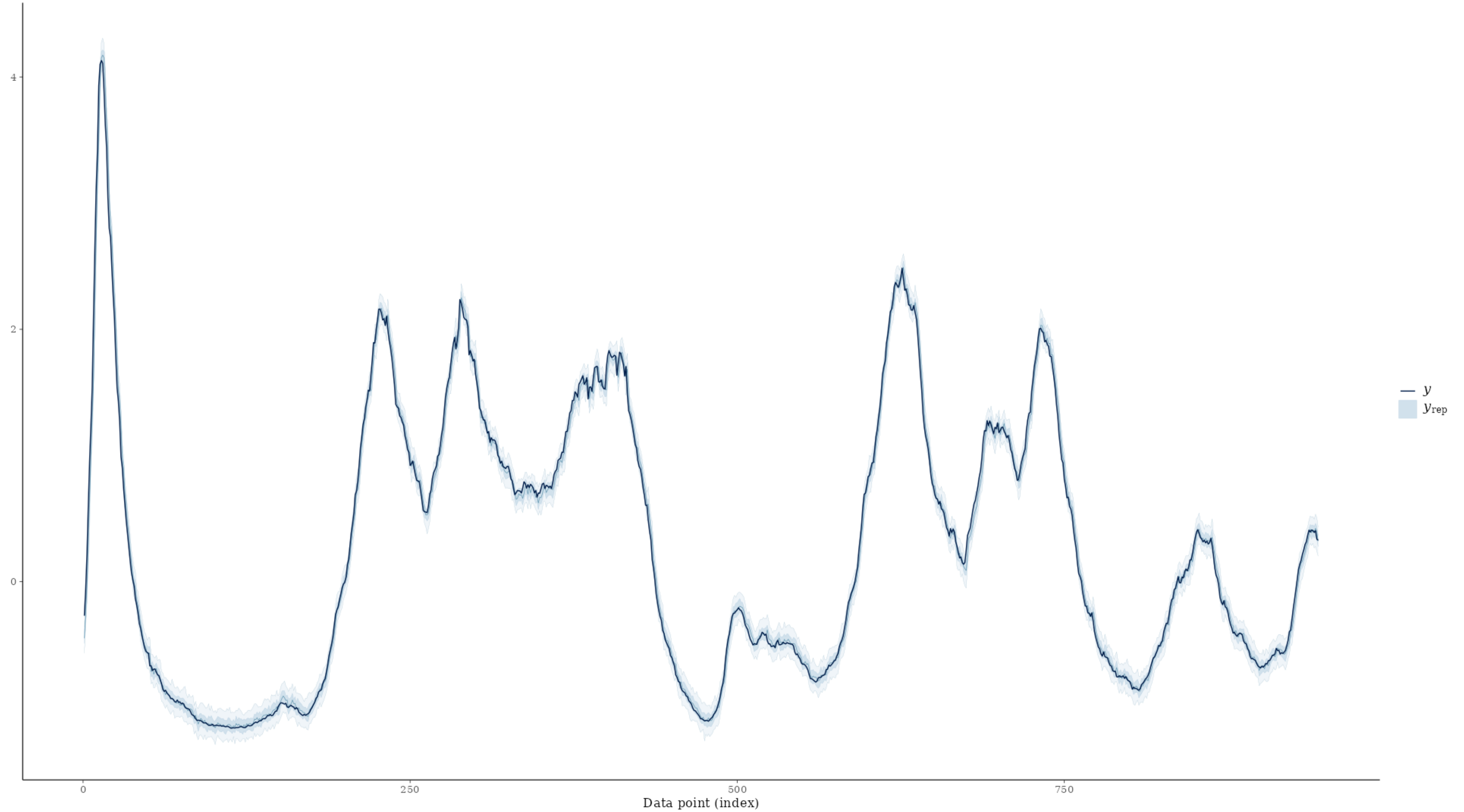

Figure A4.5 – Visits to essential shops

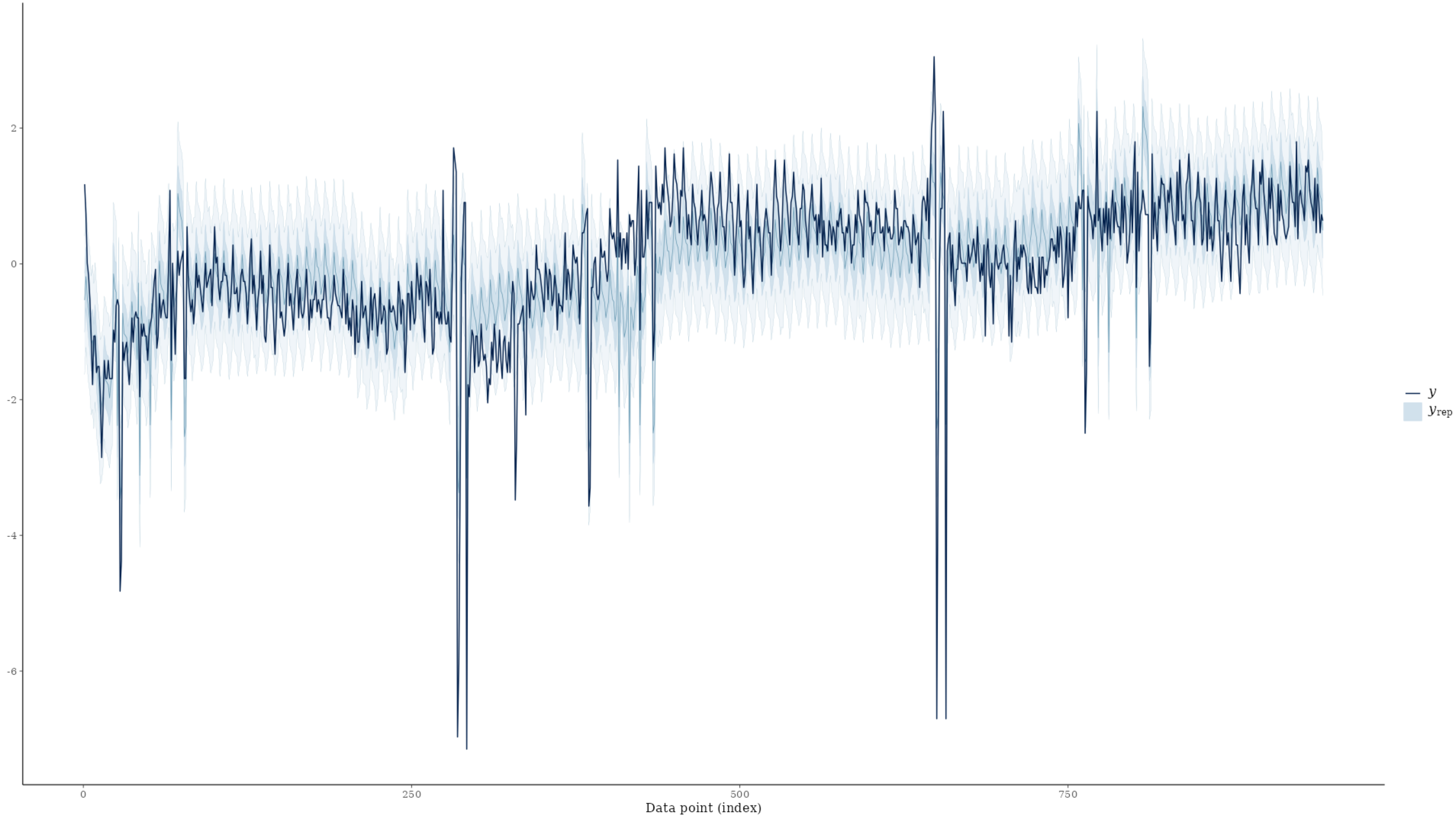

Figure A4.6 – Hospitalisations (Essential shops model)

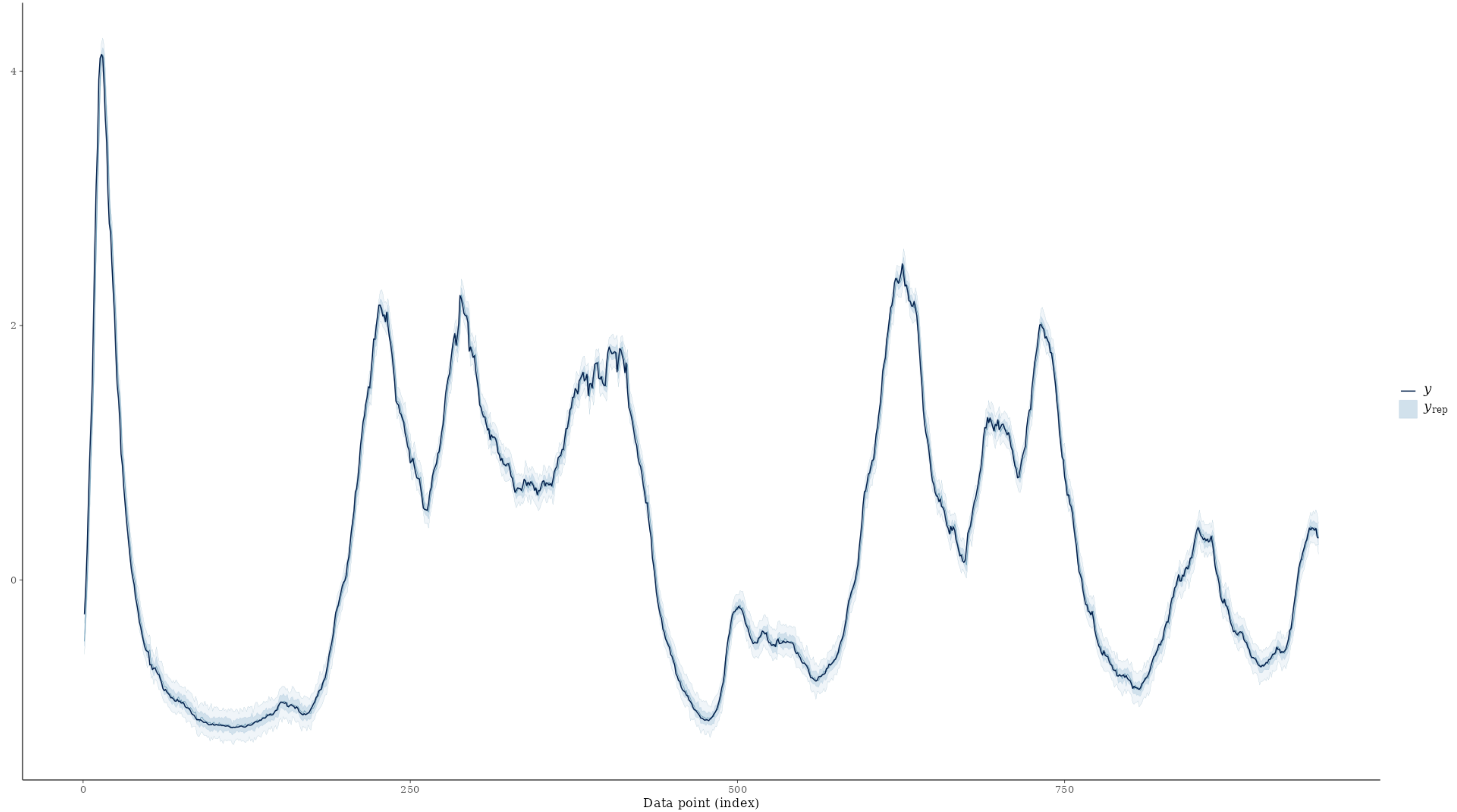

Supplementary Material part 3 – Sensitivity Analyses

This supplementary material is hosted by Eurosurveillance as supporting information alongside the article "Disentangling Enforced and Voluntary Behaviour Change During the COVID-19 Pandemic in the Netherlands", on behalf of the authors, who remain responsible for the accuracy and appropriateness of the content. The same standards for ethics, copyright, attributions and permissions as for the article apply. Supplements are not edited by Eurosurveillance and the journal is not responsible for the maintenance of any links or email addresses provided therein."

Sensitivity analysis 1: Removing Christmas and New-Year holiday period (survey data)

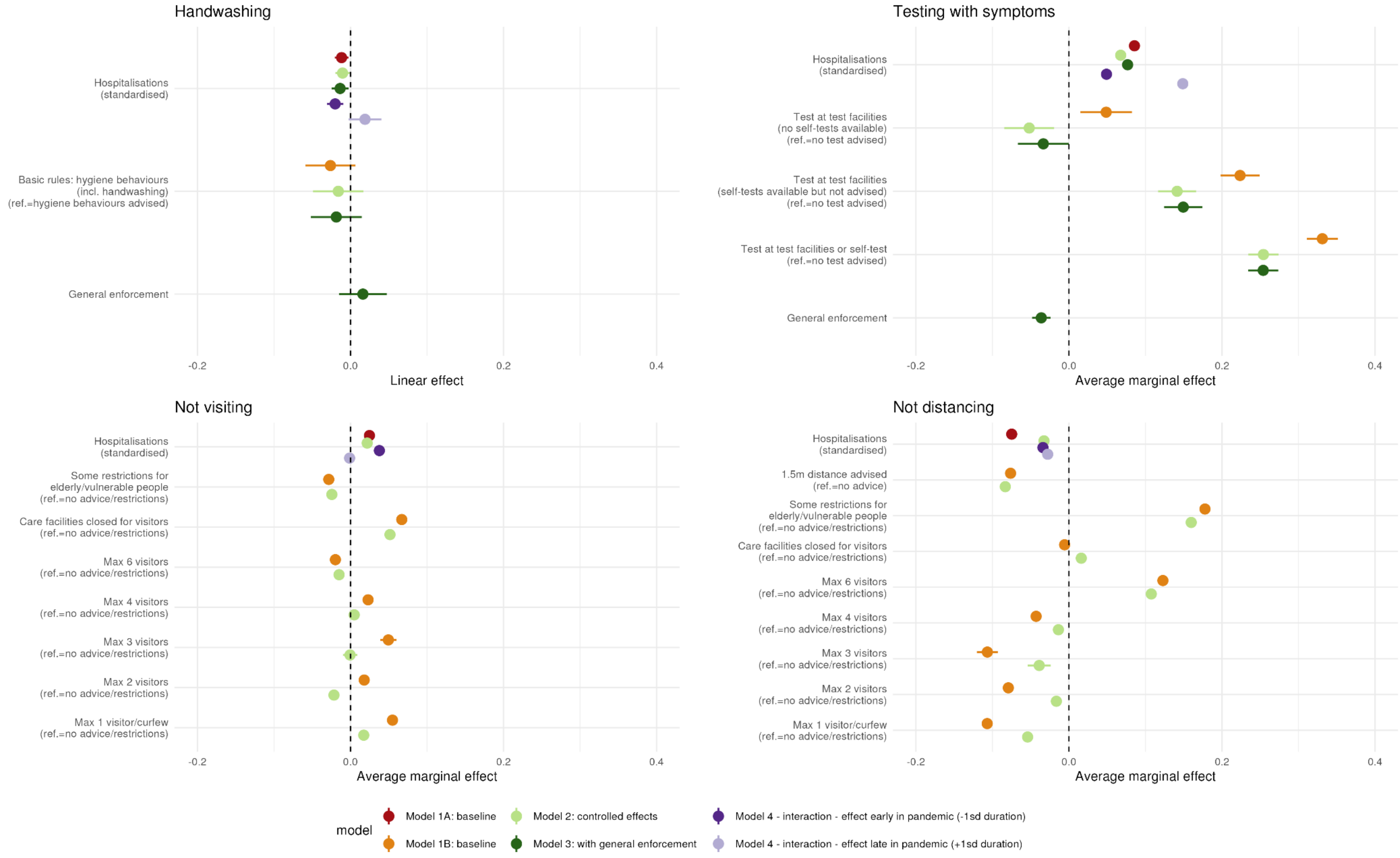

Sensitivity analysis 2A: Alternative measure of hospitalisations – 1 week after behaviour-date (survey data)

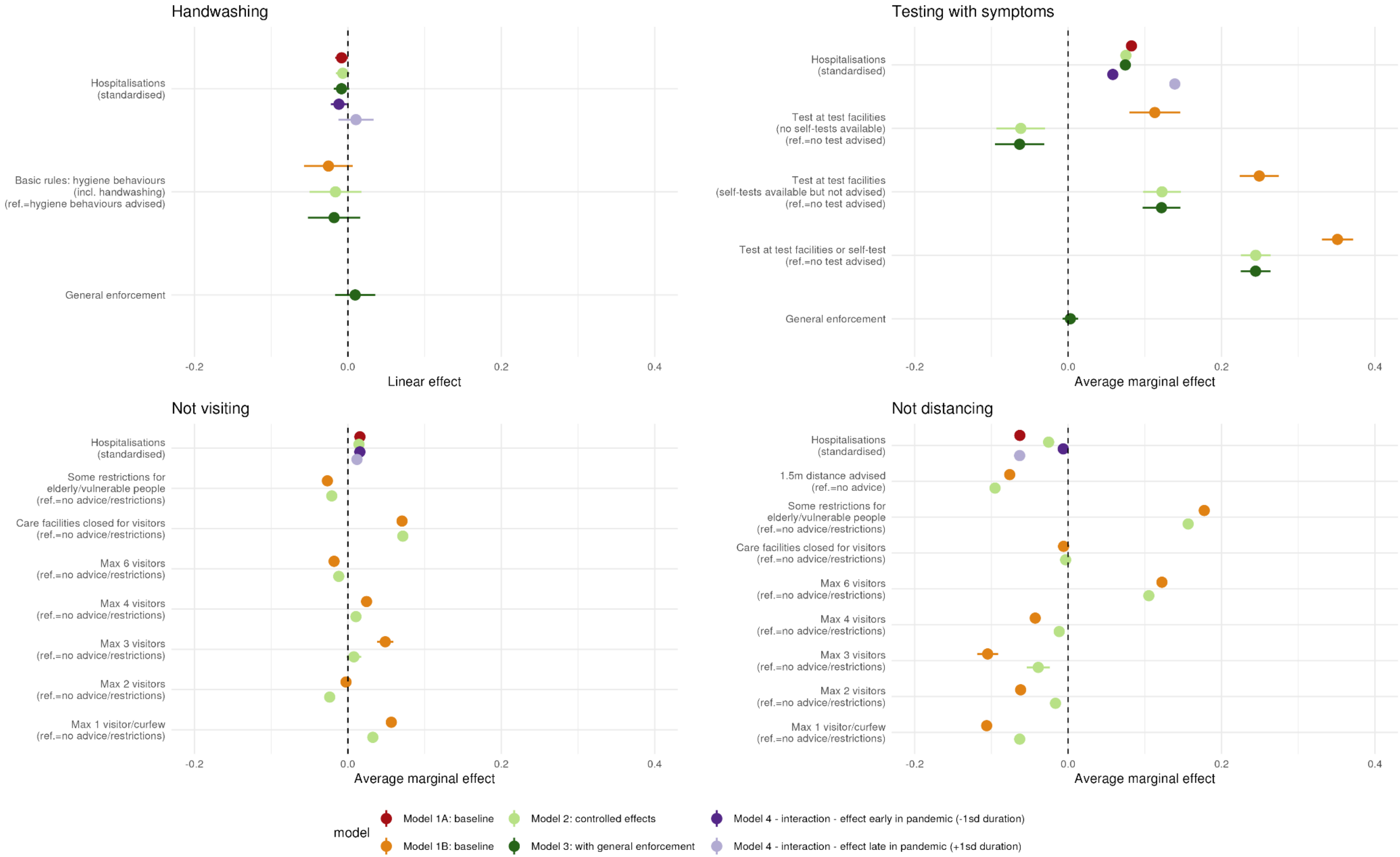

Sensitivity analysis 2B: Alternative measure of hospitalisations – 2 weeks after behaviour-date (survey data)

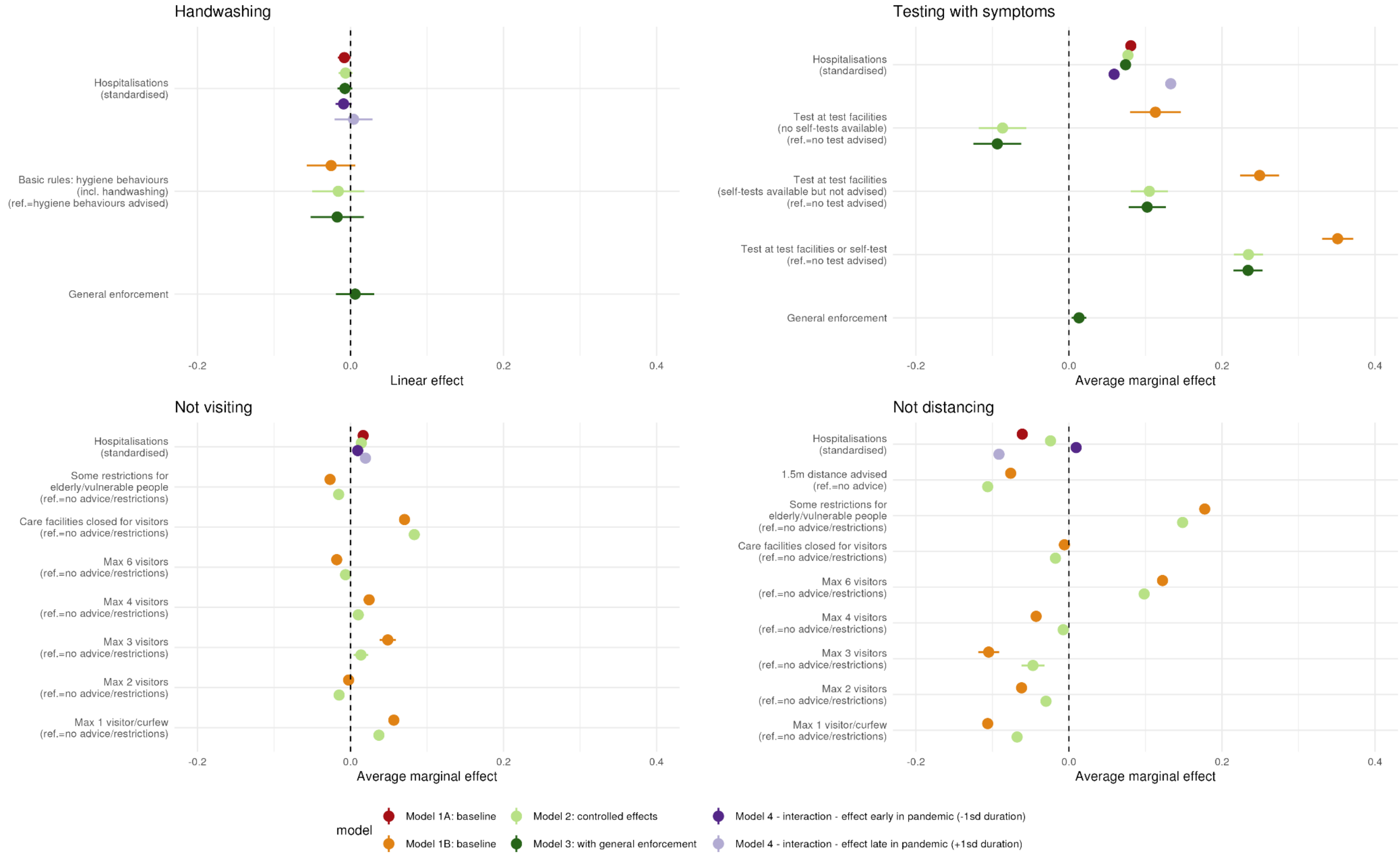

Sensitivity analysis 2C: Alternative measure of hospitalisations – 3 weeks after behaviour-date (survey data)

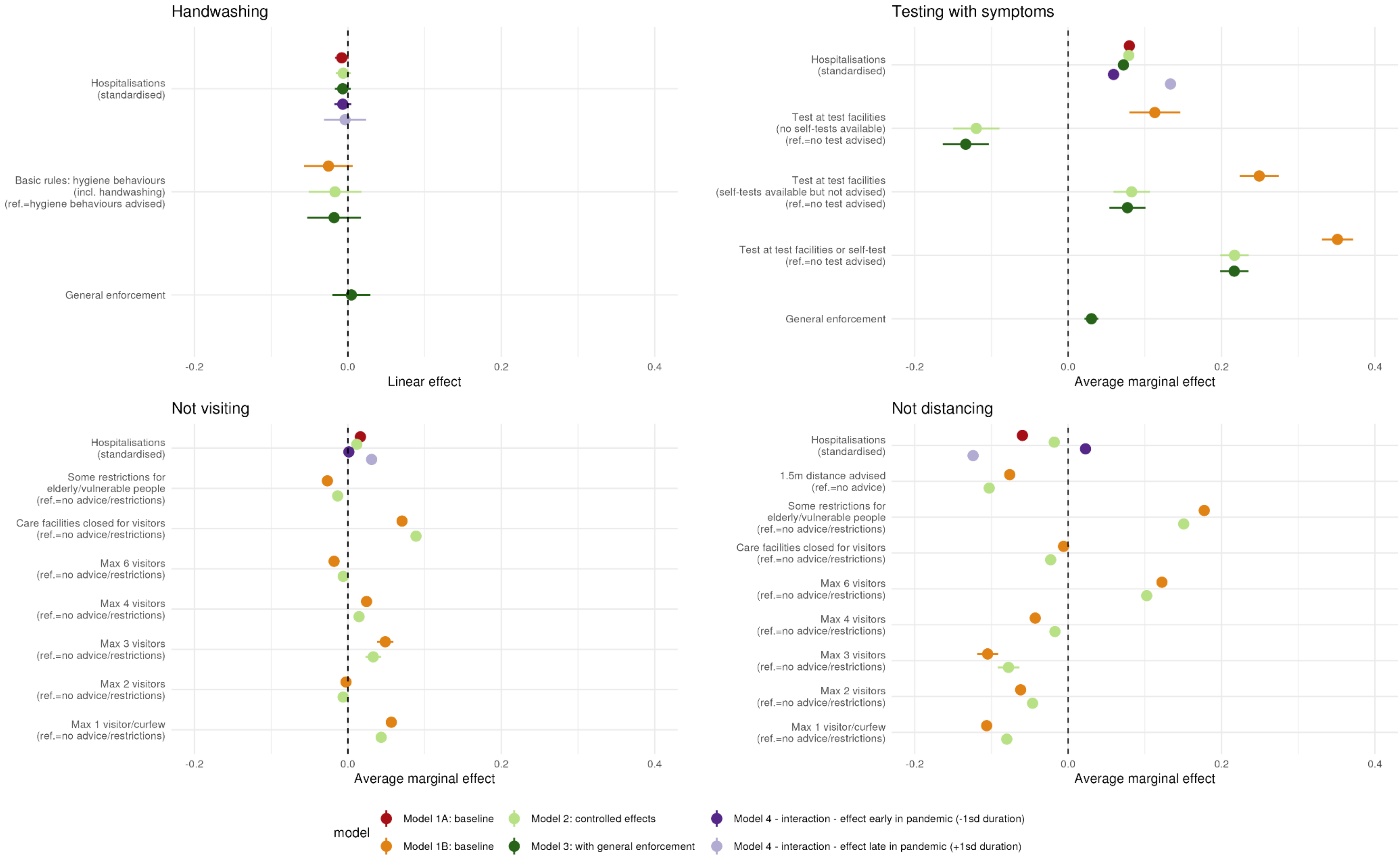

Sensitivity analysis 3: Distinction between pre- and post Omicron-wave for pandemic severity (survey data)

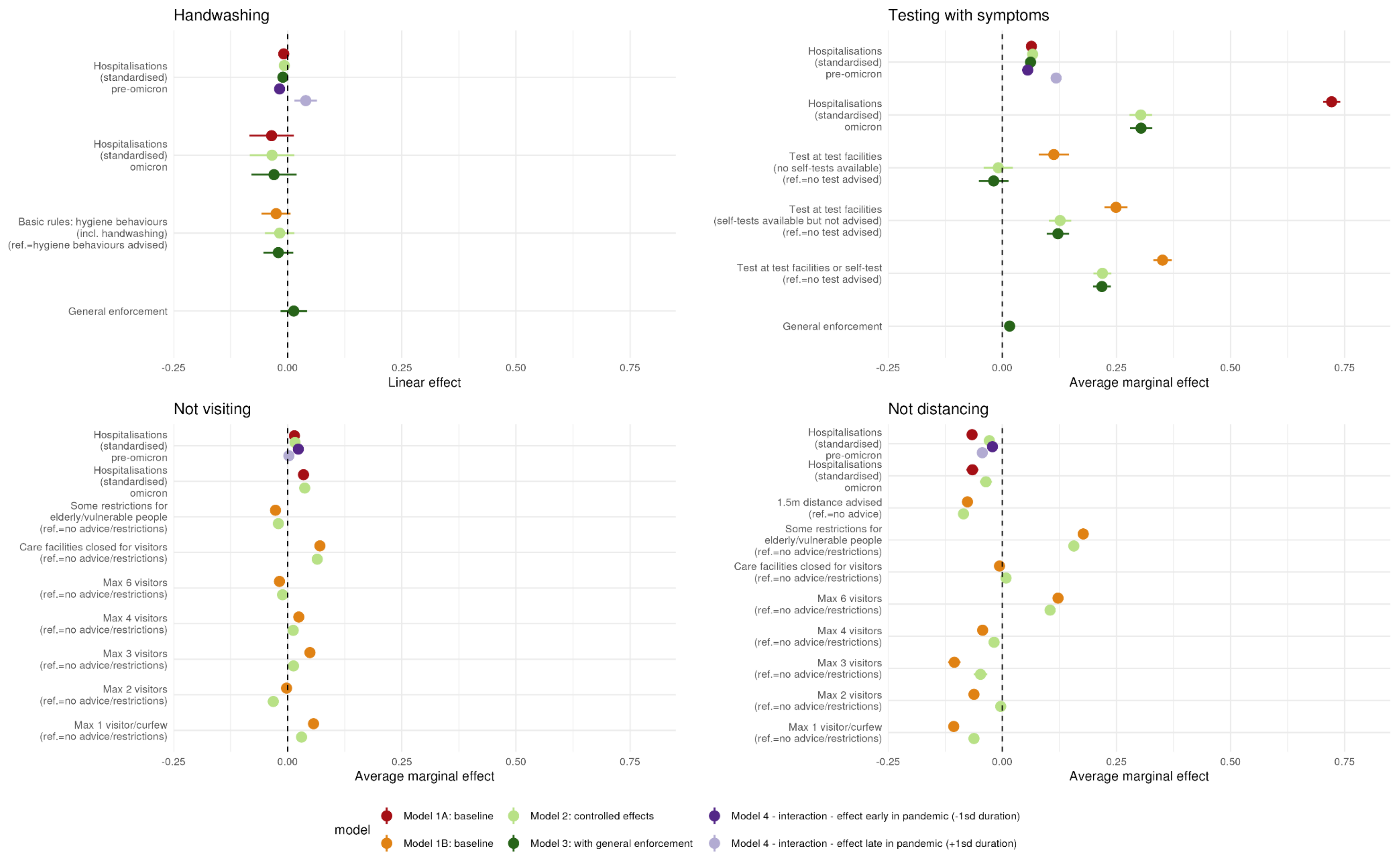

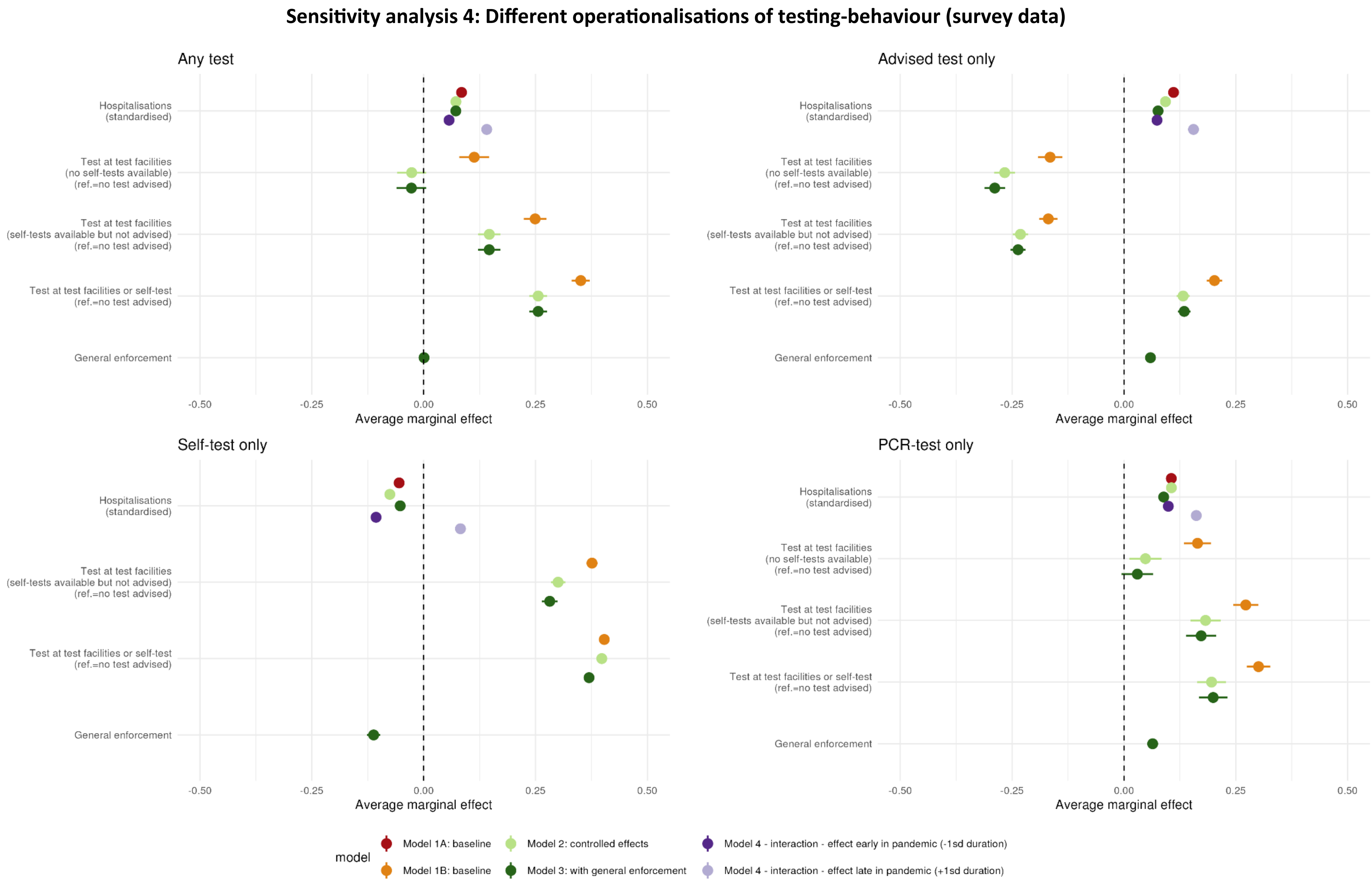
Sensitivity analysis 4: Different operationalisations of testing-behaviour (survey data)
Any test
Advised test only
Self-test only
PCR-test only
Hospitalisations (standardised)
Test at test facilities (no self-tests available) (ref.=no test advised)
Test at test facilities (self-tests available but not advised) (ref.=no test advised)
Test at test facilities or self-test (ref.=no test advised)
General enforcement
-0.50
-0.25
0.00
0.25
0.50
Average marginal effect
model
Model 1A: baseline
Model 1B: baseline
Model 2: controlled effects
Model 3: with general enforcement
Model 4 - interaction - effect early in pandemic (-1sd duration)
Model 4 - interaction - effect late in pandemic (+1sd duration)

Sensitivity analysis 5A: Alternative measure of hospitalisations – 1 week after behaviour-date (mobility data, Model 4)

Figure R5A.1 – Work mobility

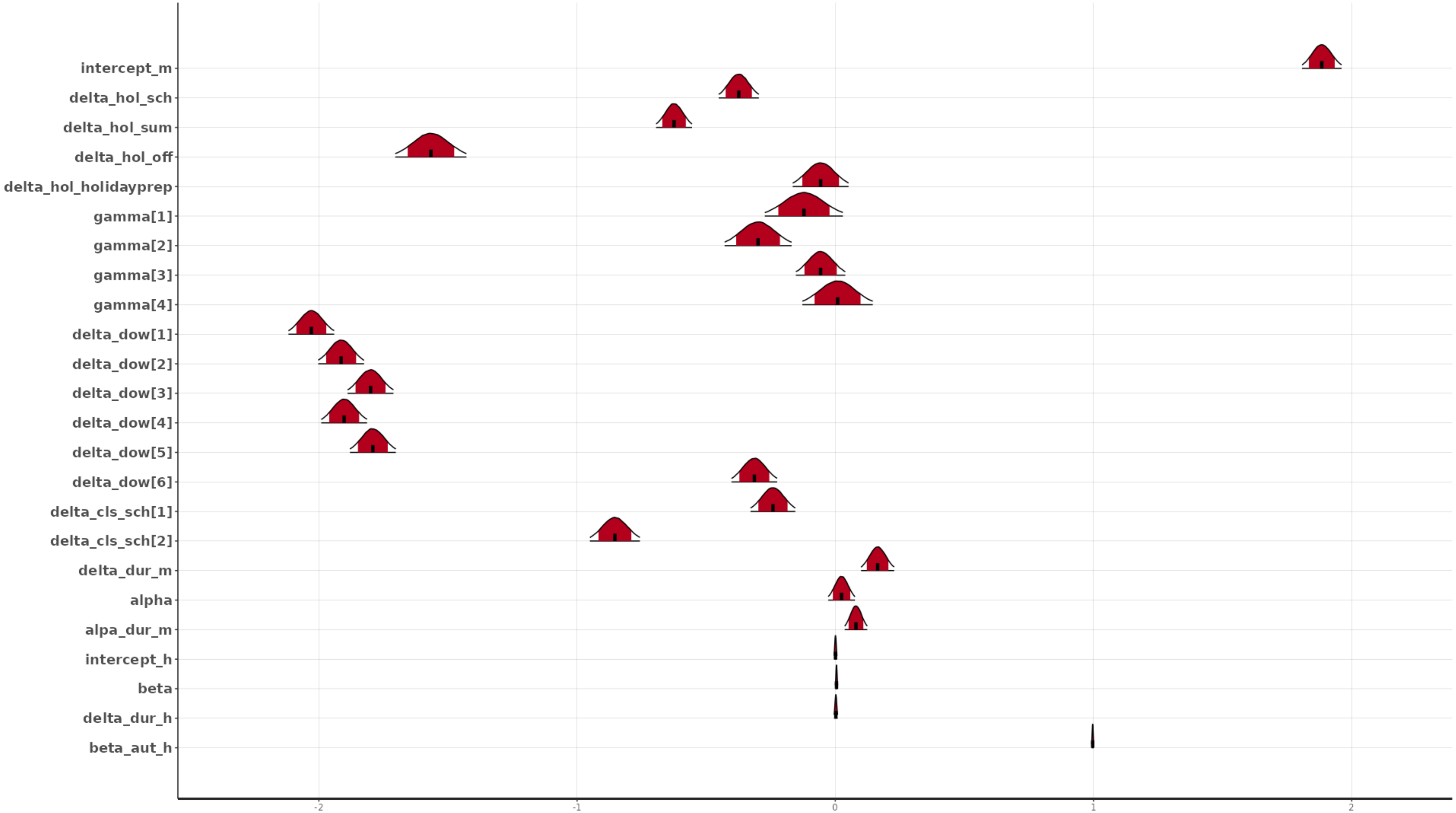

Figure R5A.2 – Recreational mobility

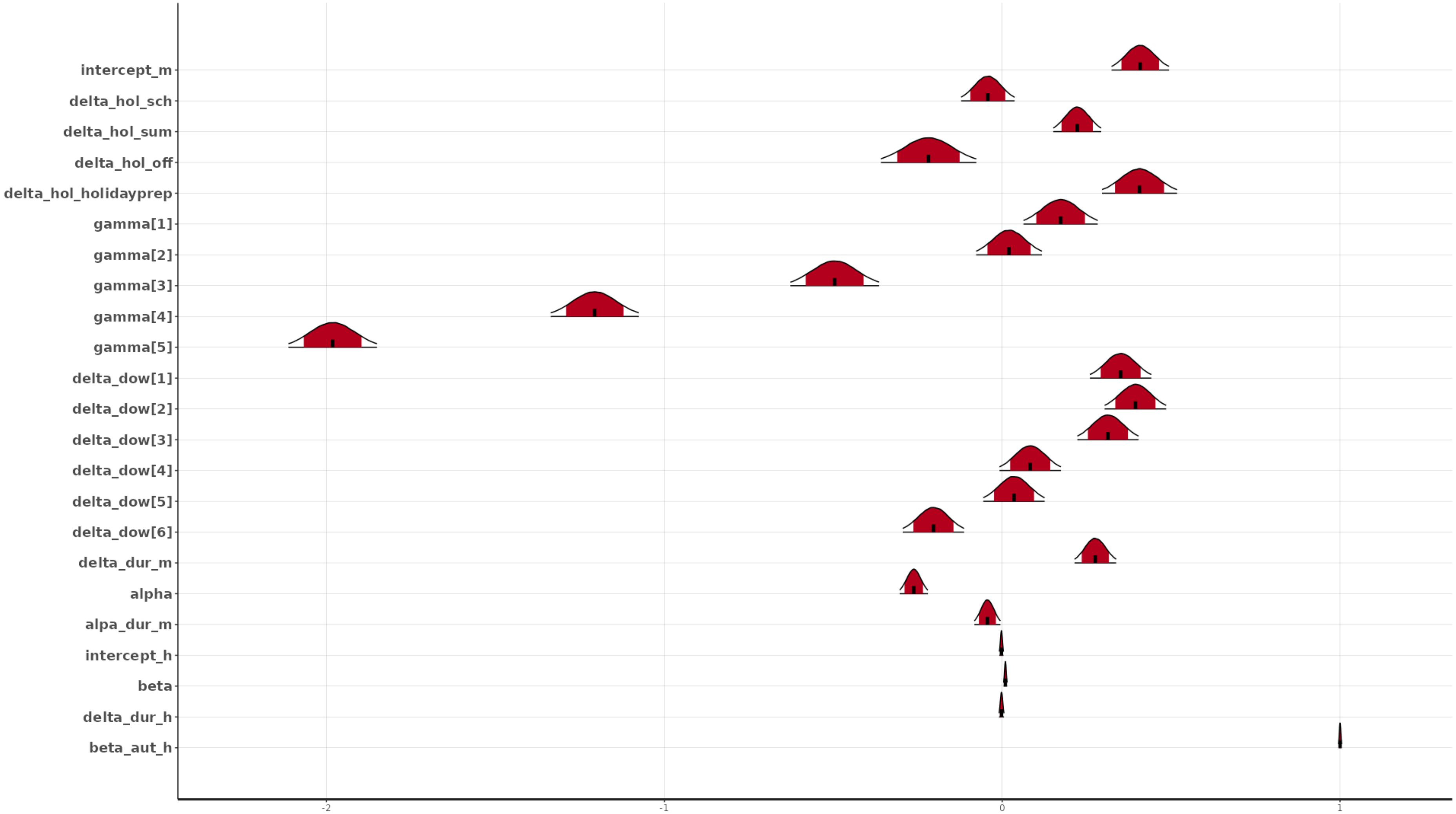

Figure R5A.3 – Supermarkets and pharmacies

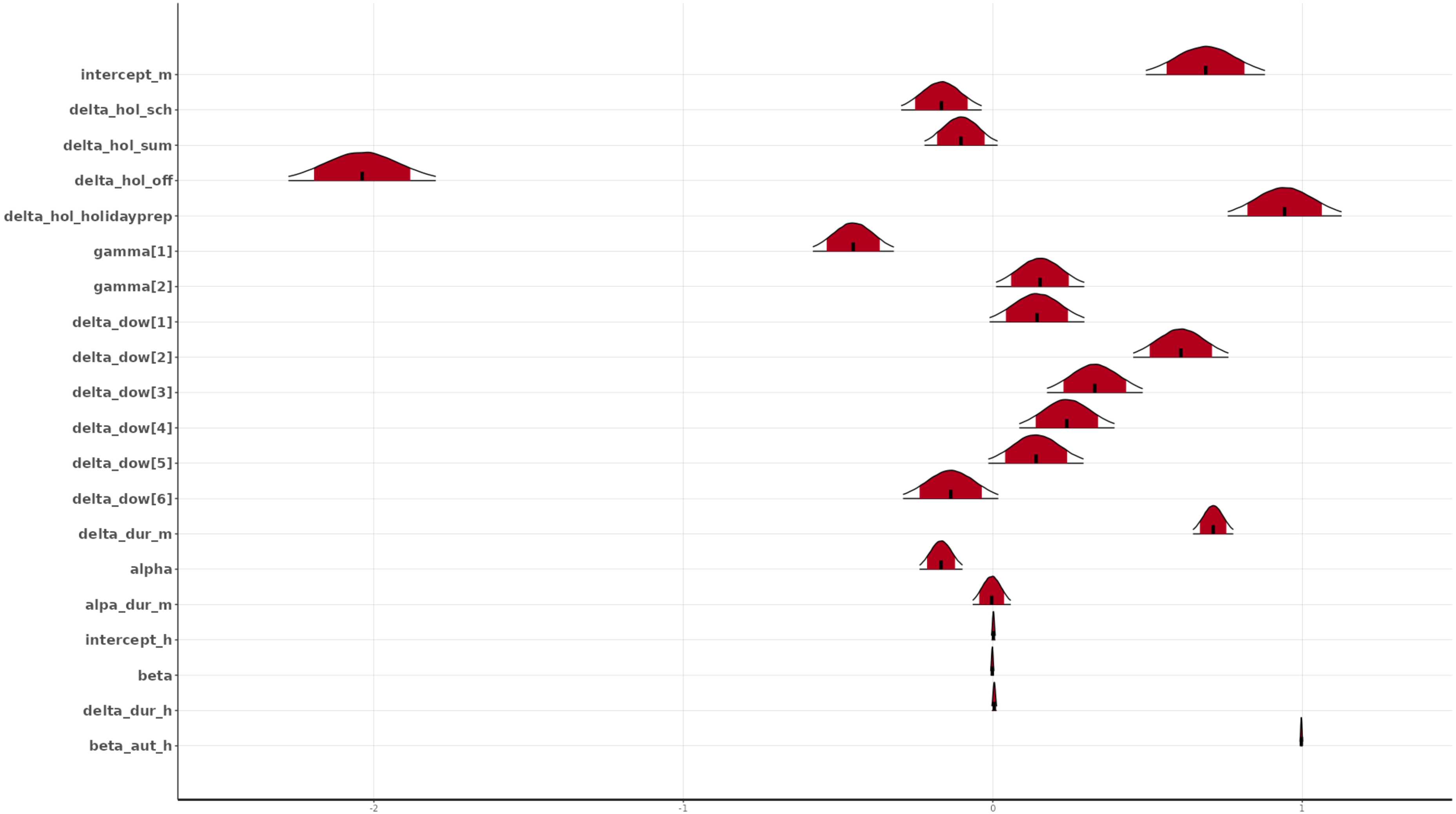

Sensitivity analysis 5B: Alternative measure of hospitalisations – 2 weeks after behaviour-date (mobility data, Model 4)

Figure R5B.1 – Work mobility

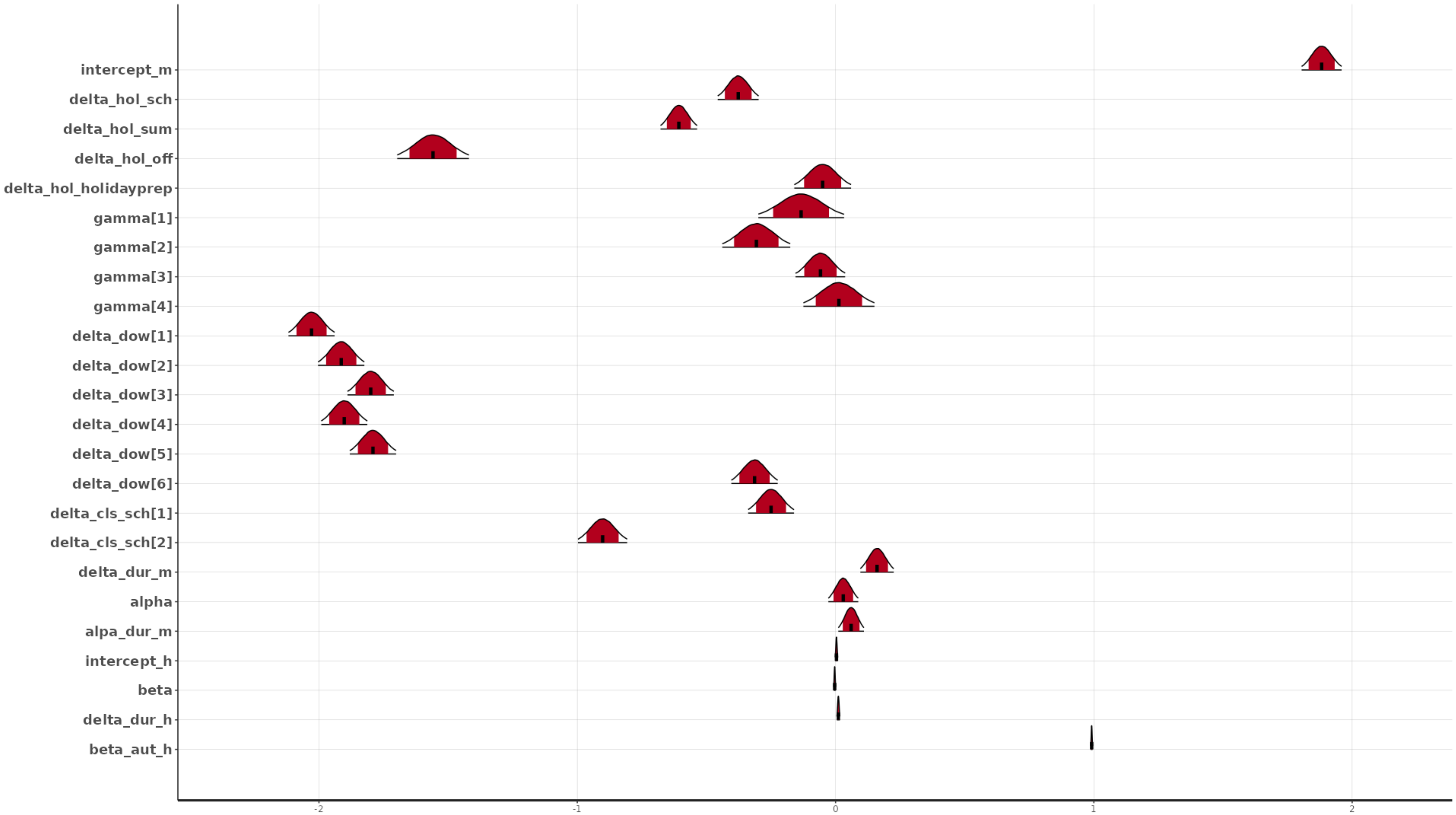

Figure R5B.2 – Recreational mobility

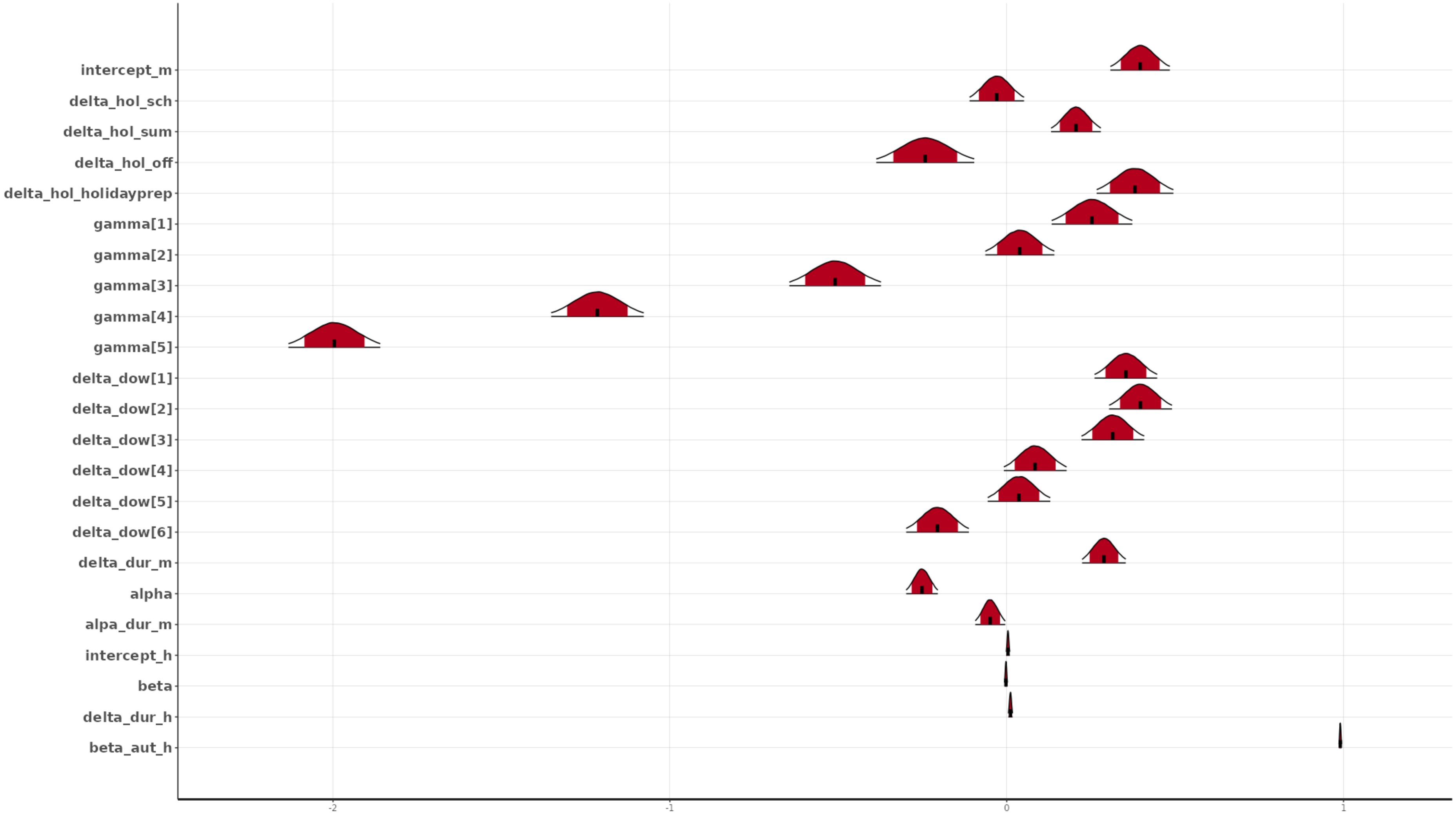

Figure R5B.3 – Supermarkets and pharmacies

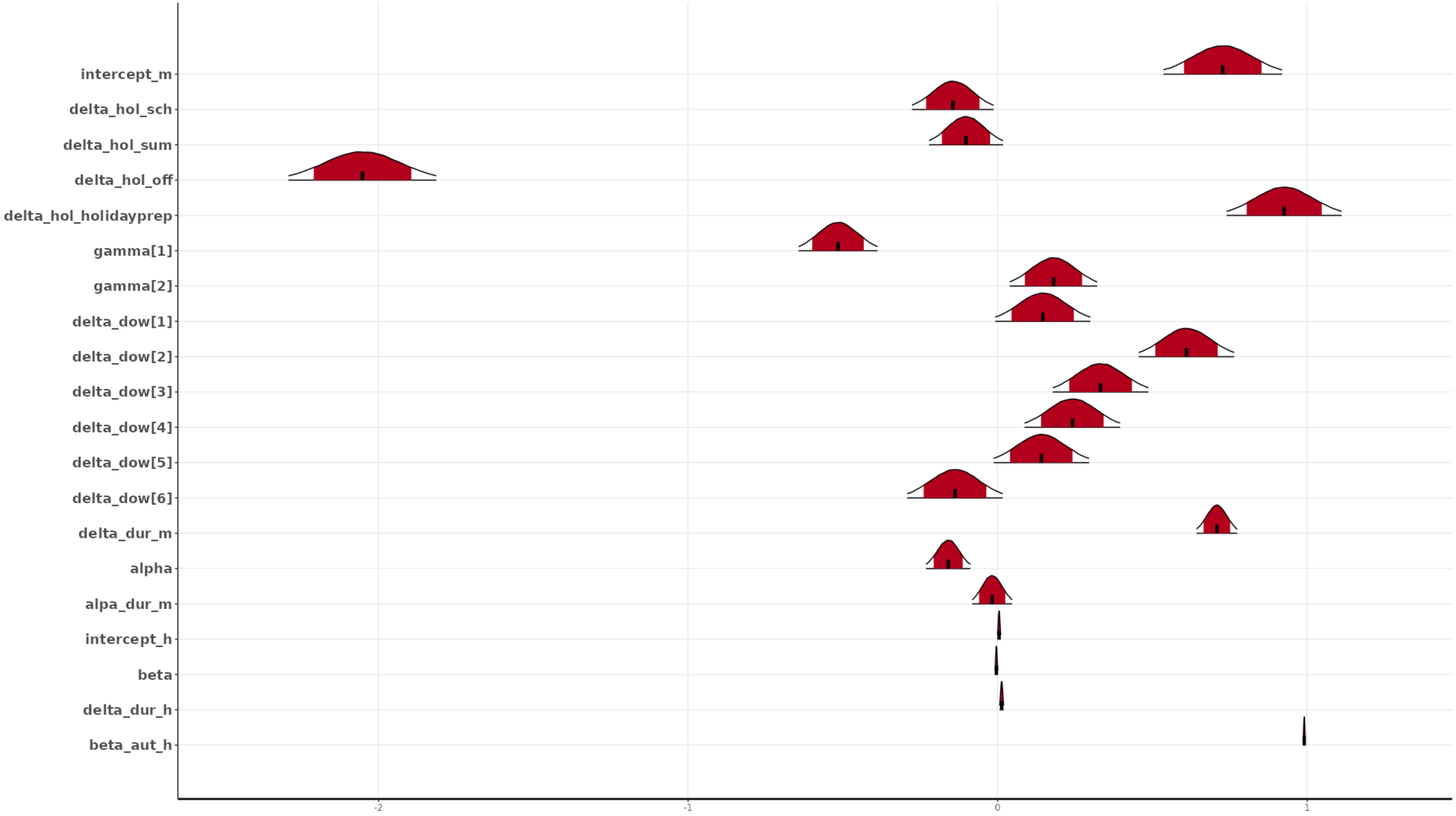

Sensitivity analysis 5C: Alternative measure of hospitalisations – 3 weeks after behaviour-date (mobility data, Model 4)

Figure R5C.1 – Work mobility

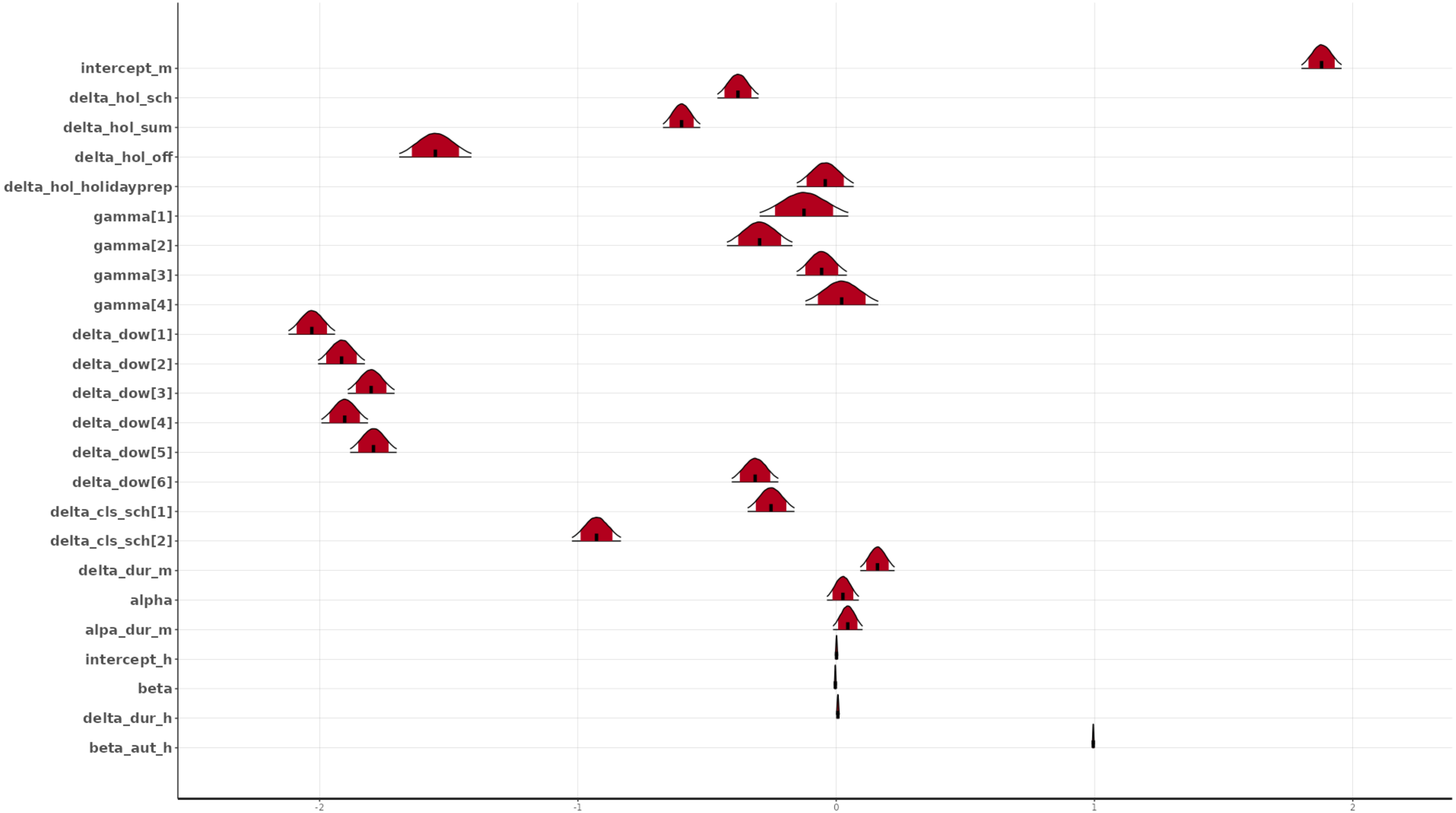

Figure R5C.2 – Recreational mobility

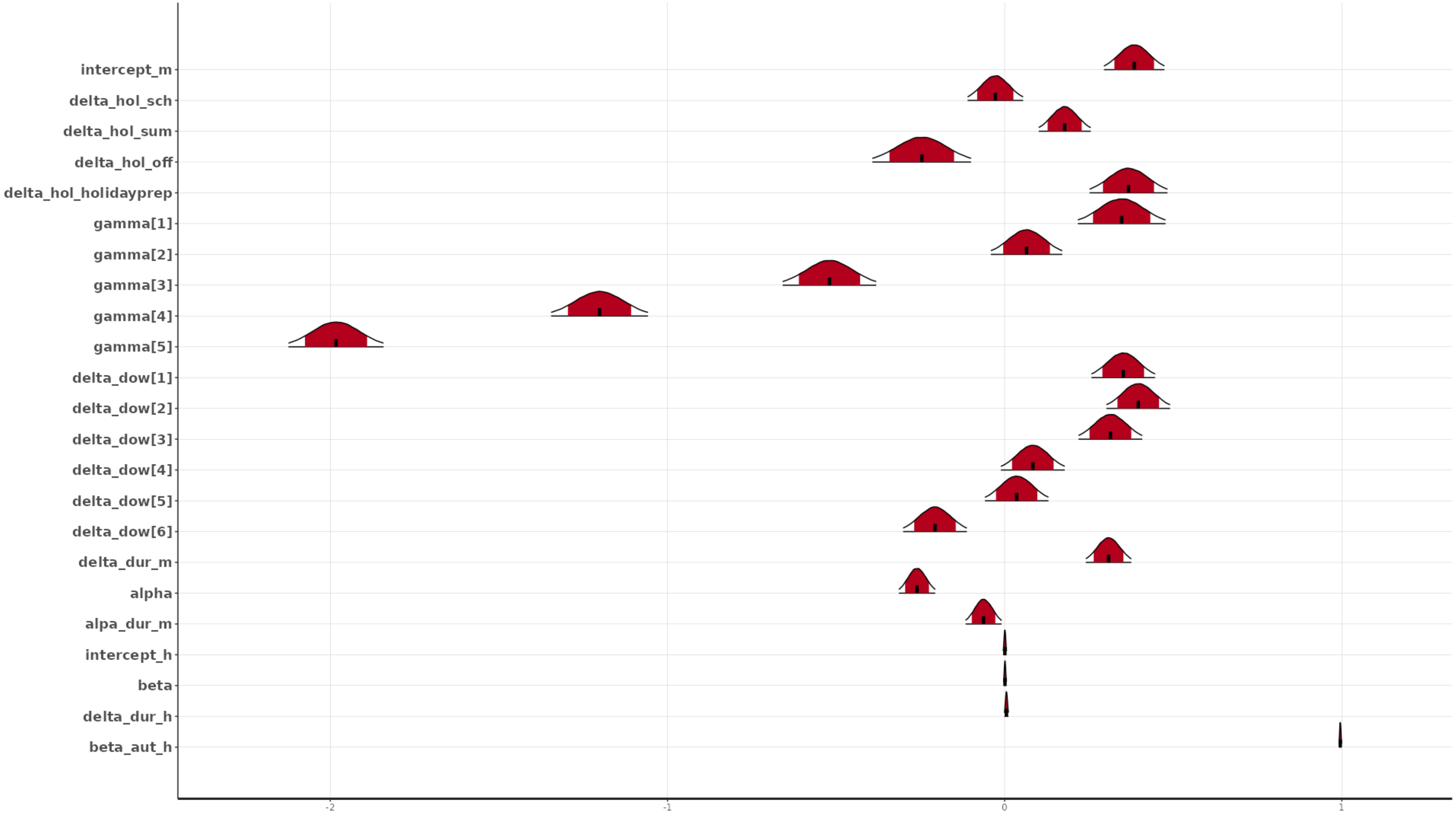

Figure R5C.3 – Supermarkets and pharmacies

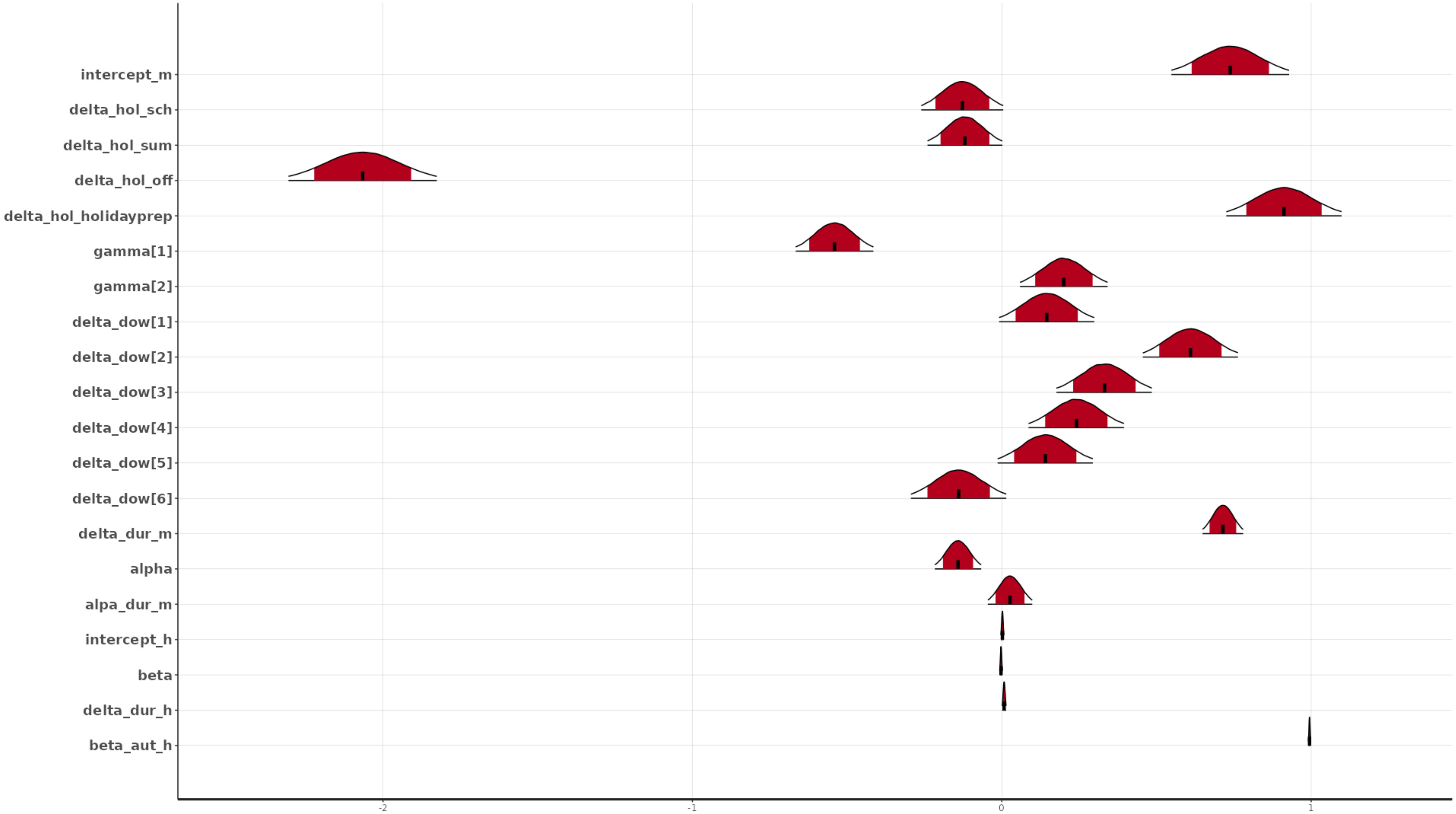

Exploratory analysis: Including advice or mandate to wear a face mask in indoor public spaces [Gamma 6/7 and 3/4] (mobility data, Model 4)

Figure R6.1– Recreational mobility [Gamma 6/7]

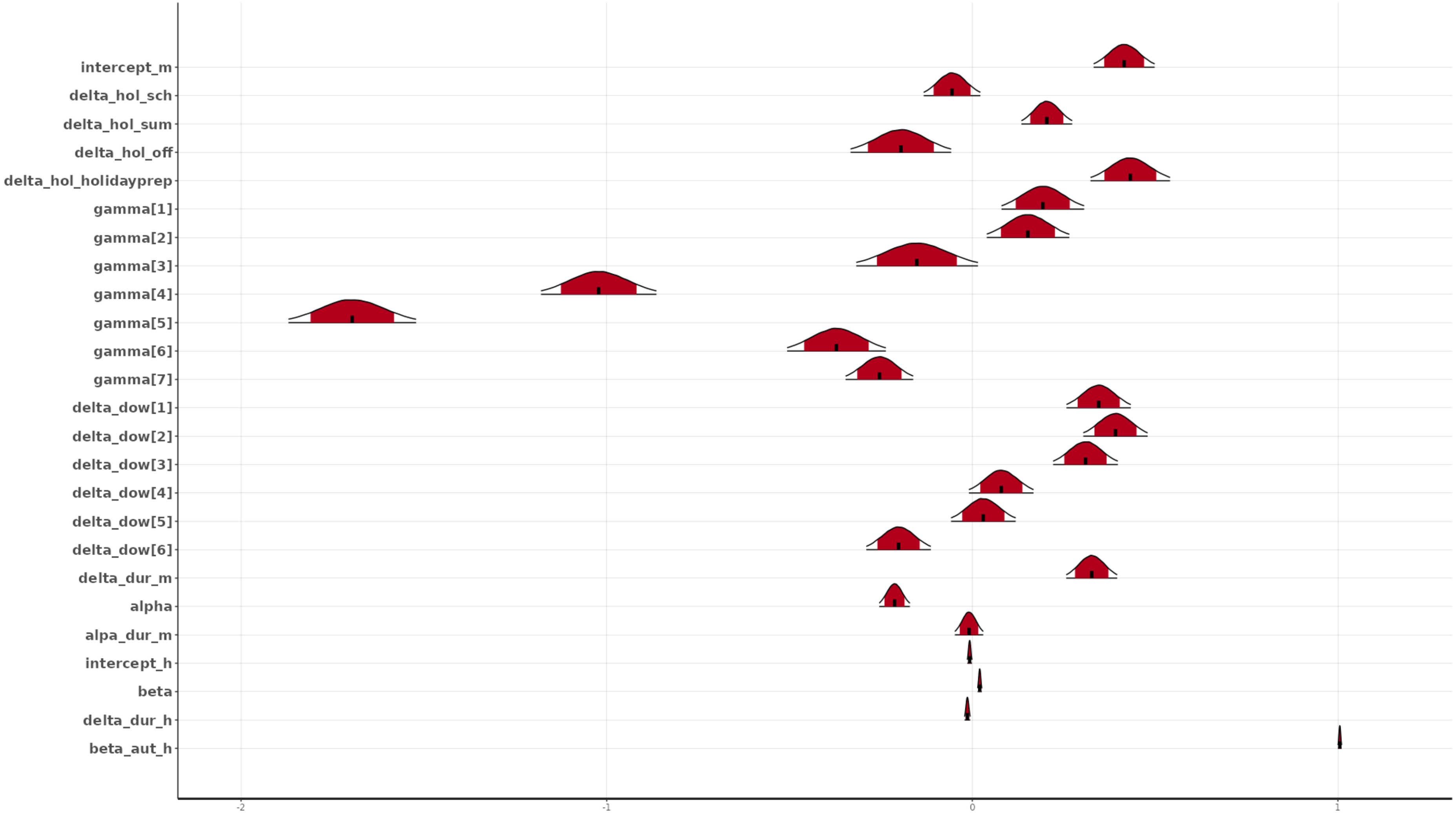

Figure R6.2 – Supermarkets and pharmacies [Gamma 3/4]

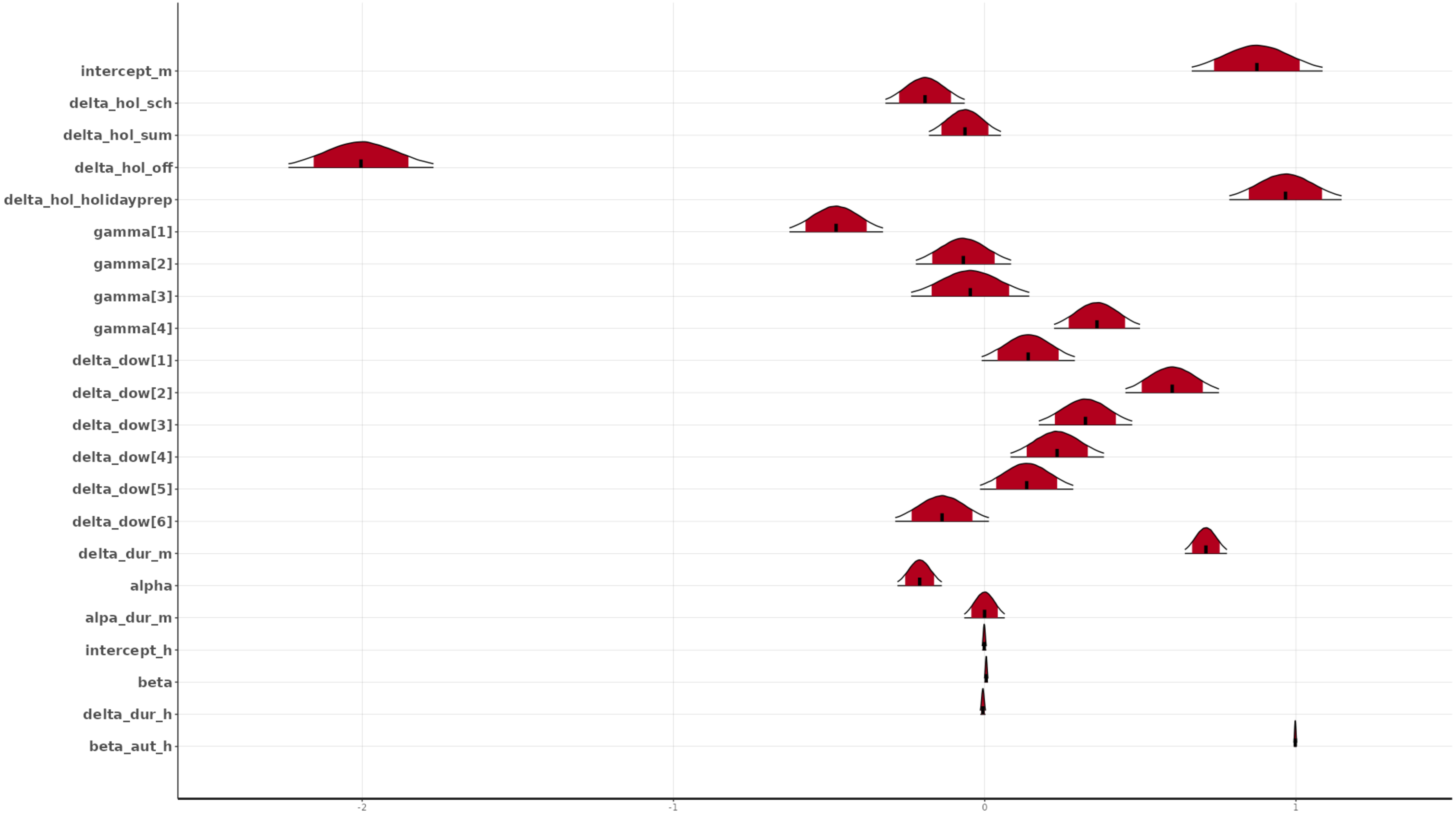

Supplement: Supplementary file 1 [file 26-00078_GIARDINA_Supplement.pdf]
